# Qifuyin alleviates anxiety and depression in 3×Tg-AD mice by modulating neuroendocrine function

**DOI:** 10.3389/fpsyt.2025.1554866

**Published:** 2025-05-14

**Authors:** Tianhao Yu, Ying Yu, Junqi Zhao, He Li, Hui Lu, Yangyi Li, Yuqi Peng, Shixue Wang, Wendi Wei, Xiaorui Cheng

**Affiliations:** Institute of Innovation in Traditional Chinese Medicine, Shandong University of Traditional Chinese Medicine, Jinan, China

**Keywords:** Alzheimer’s disease, anxiety, depression, neuroendocrine, traditional Chinese medicine prescription, Qifuyin

## Abstract

**Background:**

Alzheimer’s disease (AD) is frequently accompanied by behavioral and psychological symptoms of dementia (BPSD). Studies have shown that 3×Tg-AD mice, a classical animal model of AD, exhibit anxiety and depression-like behaviors characteristic of BPSD.

**Objective:**

This study investigated the effects of Qifuyin on anxiety and depression-like behaviors in 3×Tg-AD mice.

**Methods:**

The 20 male and female C57BL/6 mice at 10.3 months of age were used as the control group, while the 82 male and female 3×Tg-AD mice of the same age were divided into five groups. The control and model groups were gavaged with solvent, the positive medicine group received a combination of donepezil and memantine, and the Qifuyin (QFY) groups were divided into three doses: low, medium, and high. The effects of QFY on anxiety-like behaviors in mice were assessed using the open field test (OFT) and elevated plus maze (EPM) test, while depression-like behaviors were evaluated through the forced swim test (FST) and sucrose splash test (ST). Plasma levels of corticosterone (CORT), testosterone (T), and estradiol (E2) were measured using ELISA, while adrenocorticotropic hormone (ACTH), follicle-stimulating hormone (FSH), luteinizing hormone (LH), corticotropin-releasing hormone (CRH), and gonadotropin-releasing hormone (GnRH) were quantified via radioimmunoassay. Differences in plasma hormone levels among groups were analyzed using principal component analysis (PCA). Pearson correlation analysis was conducted to explore the relationships between plasma hormones and behavioral phenotypes, and multiple linear regression was employed to identify the hormones most strongly correlated with anxiety and depression-like behaviors in mice following QFY treatment.

**Results:**

In 3×Tg-AD mice, anxiety-like behaviors were characterized by reduced the duration, number of visits, and total distances in central area during the OFT. The EPM revealed reduced the duration and frequency in the open arms for both sexes. Depression-like behaviors were evident in the FST, with increased immobility, and in the ST, with prolonged grooming latency in both sexes and reduced grooming frequency in females. The treatment of QFY alleviated these behaviors. In males, In the model group, plasma ACTH, GnRH, and FSH levels were significantly decreased. In the QFY-treated group, plasma CRH levels were significantly reduced, while GnRH levels were significantly increased. In the model group of females, plasma ACTH levels were significantly elevated, while FSH and LH levels were markedly reduced. In the QFY-treated group, plasma CORT levels were significantly decreased, whereas FSH and LH levels were significantly increased. Multiple linear regression indicated QFY mainly mitigates anxiety and depression-like symptoms through modulating GnRH in males and T and ACTH in females.

**Conclusions:**

The administration of QFY alleviates anxiety and depression in 3×Tg-AD mice by regulating the HPA, HPT and HPO axes.

## 1 Introduction

Alzheimer’s disease (AD) is a progressive neurodegenerative disorder and the most prevalent cause of dementia (1). It is primarily characterized by a decline in memory and the ability to perform daily activities (2). Studies suggest that between 56% and 98% of individuals with AD develop behavioral and psychological symptoms of dementia (BPSD) (3–5), which encompass a range of issues including depression, apathy, agitation, repetitive questioning, psychosis, aggression, and sleep disturbances (5, 6). Early signs of AD often manifest as apathy and anxiety (7), with approximately 40% of patients exhibiting anxiety symptoms (8) and 30% to 50% suffering from comorbid depression (9, 10). The presence of these symptoms not only increases the stress on caregivers but also contributes significantly to the societal burden (4, 11–15). The pathophysiology of AD is complex and is associated with an increased risk of anxiety and depression, which can precede memory impairment in the early stages of the disease. Anxiety and depression have been observed during the mild cognitive impairment (MCI) phase, which often precedes AD (16–19), and anxiety has been shown to heighten the risk of MCI patients progressing to dementia (20).

The etiology of BPSD is multifaceted, with research indicating associations with elevated levels of the G9a epigenetic enzyme, deficits in autophagy, and synaptic loss (21, 22). A meta-analysis has highlighted the role of serotonin (5-HT) and its receptors, particularly the 5-HT2A receptor, are linked to depressive, delusional, and agitated moods in BPSD (23, 24). Additionally, many evidence points to neuroendocrine dysregulation, particularly involving the hypothalamic-pituitary-adrenal(HPA) axis (25, 26). While a direct link between hypothalamic-pituitary-thyroid(HPT) axis, hypothalamic-pituitary-ovarian(HPO) axis dysfunction and BPSD has been suggested, there is a clear association between HPT, HPO axis dysregulation and the development of anxiety and depression (27–29). The HPA axis governs stress responses via CRH-ACTH-CORT signaling, and chronic hyperactivity elevates cortisol, exacerbating AD pathology through hippocampal atrophy, neuroinflammation, and impaired serotonin/dopamine neurotransmission (30, 31). Elevated cortisol levels correlate with accelerated cognitive decline severity in AD patients (32). Research shows that anxiety and stress can lead to the activation of the HPA axis (33–35). Dysfunction of the HPO and HPT axes has been implicated in the pathogenesis of anxiety and depression (36). Studies have shown that GnRH antagonists can induce anxiety- and depression-like behaviors (37, 38), whereas testosterone has been found to exert anxiolytic and antidepressant effects (39, 40) Furthermore, growing evidence suggests that HPG axis dysfunction is associated with the development of AD. The regulatory hormones of the HPG axis—estrogen in females and testosterone in males—play neuroprotective roles by inhibiting β-amyloid (Aβ) deposition (41). Postmenopausal estrogen deficiency exacerbates amyloid−β pathology and impairs cognitive function in women (42), while testosterone deficiency in men reduces the expression of neprilysin, an enzyme responsible for Aβ clearance, thereby increasing AD risk (43). These findings highlight the critical role of HPA, HPT and HPO axis dysregulation in both AD pathology and behavioral and psychological symptoms of dementia (BPSD), suggesting that hormonal modulation may serve as a potential therapeutic strategy for both neuropsychiatric symptoms and disease progression in AD.

The clinical management of BPSD predominantly relies on psychotropic medications (44), which can be categorized based on their action on dopamine and serotonin receptors. Typical (first-generation) antipsychotic drugs, such as chlorpromazine, haloperidol, and sulpiride, primarily exert their effects by antagonizing dopamine D2 receptors (45). In contrast, atypical (second-generation) antipsychotic drugs, including clozapine, risperidone, olanzapine, quetiapine, and aripiprazole, exert their effects through a variety of mechanisms, including the modulation of serotonin (5-HT), norepinephrine, or histamine neurotransmission (46). However, only two drugs are strictly approved for the treatment of BPSD: pimavanserin, which is approved for the treatment of hallucinations and delusions associated with Parkinson’s disease in the United States, and risperidone, which is approved for the treatment of persistent aggressive behavior in AD in Canada and Europe (47). Other medications used to treat BPSD are prescribed off-label. It is crucial to recognize that both first- and second-generation antipsychotic drugs are associated with numerous adverse effects (48) and have the potential to increase the risk of Parkinsonism, gait disturbances, cerebrovascular adverse events, cognitive decline, and even death (49–51). Natural medicines, with their multi-target properties and generally fewer adverse effects, have emerged as potential treatment options for BPSD (52). A meta-analysis (53) of four trials involving 1,628 patients found that treatment with the ginkgo extract EGb 761^®^ led to improvements in BPSD symptoms. Another prescription, Yi Gan San, is commonly used for BPSD treatment in Japan and has shown promising effects (54). In China, traditional Chinese medicine formulas are frequently employed to treat BPSD (55), frequently in combination with chemical drugs.

Qifuyin (QFY), a traditional Chinese medicine prescription, originates from “Jingyue Quanshu”(a comprehensive medical compendium written by Zhang Jingyue in the Ming Dynasty. It systematically summarizes and expands upon traditional Chinese medical theories, particularly in internal medicine and herbal prescriptions), and is composed of a blend of medicinal herbs including ginseng, cooked rehmannia, angelica sinensis, atractylodes macrocephala, honey-fried licorice, jujube seed and polygala tenuifolia. This formula is commonly used in clinical dementia treatment (56, 57), In Jingyue Quan Shu, it is recorded that dementia is often accompanied by symptoms of anxiety, panic, and depression, and that these symptoms further exacerbate the manifestations of dementia. QFY is used for treatment (58). Modern research has found that QFY can inhibit the TLR4/NF-κB pathway, thereby reducing neuroinflammation (59), the activation of the TLR4/NF-κB pathway is associated with anxiety and depression-like symptoms (60, 61), therefore, it is inferred that QFY has therapeutic potential for depressive symptoms. Our previous research has indicated that the treatment of QFY can enhances cognitive function in APP/PS1 double transgenic mice by regulating the gut microbiome (62) and ameliorate cognitive function in 5×FAD mice by modulating immunity (63). However, the impact of QFY on psychiatric and behavioral symptoms associated with AD has not yet been extensively studied. The 3×Tg-AD mouse model, which carries three gene mutations—APP (Swedish), PS1 (M146V), and tau (P301L)—exhibits pathological features that more closely resemble those of human Alzheimer’s disease compared to other models. Due to this higher fidelity, it has been widely utilized to assess the therapeutic effects of various potential AD treatments, including chemical compounds, biologics, traditional Chinese medicine, studies have shown that 3×Tg-AD mice exhibit BPSD (64).

Our present study employed the 3×Tg-AD mice to investigate the effects of QFY on anxiety and depression symptoms associated with AD from a neuroendocrine perspective.

## 2 Materials and methods

### 2.1 The preparation of QFY

The QFY dry extract powder was purchased from Lunan Pharmaceutical Group Corporation. The specific preparation method is as follows: weigh out 3.0 kg of ginseng (*Panax ginseng C. A. Mey.*), 4.50 kg of prepared rehmannia root(*Rehmanniaglutinosa (Gaertn.) Libosch. ex Fisch. & C. A. Mey.*), 4.50 kg of angelica(*Angelica sinensis (Oliv.) Diels*), 2.50 kg of stir-fried Atractylodes macrocephala(*Atractylodes macrocephala Koidz.*), 3.0 kg of sour jujube seed(*Ziziphus jujuba* var. sp*inosa (Bunge) Hu ex H.F.Chow.*), 2.50 kg of processed polygala(*Polygala tenuifolia Willd.*), and 1.50 kg of honey-fried licorice(*Glycyrrhiza uralensis Fisch*). First, perform heat reflux on the ginseng with 60% ethanol twice, each for 1.5 hours. Filter the mixture, set aside the residue, recover the ethanol from the filtrate, and concentrate it to a relative density of 1.03-1.9 (at 60°C), and set it aside. Next, extract the volatile oil from angelica and stir-fried Atractylodes macrocephala using water distillation, collect the distilled aqueous solution in a separate container, and set aside the residue. The volatile oil ethanol solution is encapsulated with beta-cyclodextrin, dried, and pulverized for later use. Perform boiling of the residues from the above three herbs along with the remaining four herbs (prepared rehmannia root, etc.) in water twice, each time for 2 hours. Filter the mixture and mix the filtrate with the concentrated ginseng solution. Perform concentration of the mixture to a relative density of 1.02-1.06 (at 60°C) to obtain a clear syrup, let it stand, centrifuge, and then concentrate to a relative density of 1.22-1.28 (at 60°C) to obtain a dense extract. Dry and pulverize the extract, then mix it with the beta-cyclodextrin encapsulated substance.

### 2.2 Animals and treatment

The 3×Tg-AD transgenic mice (65)[strain B6;129-Tg(APPSwe,tauP301L)1Lfa Psen1 tm1Mpm/Mmjax], carrying three mutations associated with familial Alzheimer’s disease (APP Swedish, MAPT P301L, and PSEN1 M146V), were purchased from the Jackson Laboratory. The C57BL/6J mice were purchased from Beijing Huafukang Bioscience Co., Ltd. A total of 111 mice were included in the study, comprising 53 males and 58 females. Both the C57BL/6J and 3×Tg-AD transgenic mice were housed at the Experimental Animal Center of Shandong University of Traditional Chinese Medicine until they reached 10.3 months of age. All animals were maintained at a temperature of 23 ± 1°C under a 12-hour light/dark cycle with free access to food and water. Before the experiments, all mice were acclimated to the experimental environment for 6 days. All animal-related experiments have been reviewed and approved by the ethics committee of Shandong University of Traditional Chinese Medicine (Ethics No.SDUTCM202209291). All efforts were taken to minimize the number of animals used and their suffering. The 10.3-month-old C57BL/6J and 3×Tg-AD transgenic mice were divided into six groups based on activity level and body weight. Each group consisted of 8–9 mice, and the treatment was administered via oral gavage: control group(male10, female10): C57BL/6J mice, model group(male7, female7): 3×Tg-AD transgenic mice, positive drug group(male8, female9): 3×Tg-AD + donepezil (1.0 mg/kg/day) and memantine (2.8 mg/kg/day), QFY low-dose group(male8, female10): 3×Tg-AD + QFY (1.06 g/kg/day), QFY medium-dose group(male7, female10): 3×Tg-AD + QFY (2.12 g/kg/day), QFY high-dose group(male7, female9): 3×Tg-AD + QFY (4.24 g/kg/day), The control and model groups were administered distilled water by gavage for the duration of the study. All mice underwent behavioral tests following 305 days of treatment, and samples were collected for biochemical analysis after 328 days of treatment.

### 2.3 Behavioral tests

#### 2.3.1 Open field test

The open field test was performed using an open field apparatus measuring 41 cm in length × 41 cm in width × 30 cm in height to assess the exploratory activity and anxiety-like behavior of the mice (66, 67). Each mouse was gently placed at the center of the apparatus, with the central area being demarcated as a square zone 9 cm from the perimeter walls. A sophisticated video tracking system, Tracking Master V3.0 (FANBI Intelligent Technology Co., Ltd., Shanghai, China), was employed to meticulously record the time spent, the number of entries, and the distance traveled within the central area over a 9-minute interval for each mouse.

#### 2.3.2 Elevated plus maze

The elevated plus maze, with dimensions of 66.5 cm in length × 6.5 cm in width × 45.5 cm in height, was used to assess anxiety-like behavior (68, 69). The closed arms were surrounded by 15 cm high walls. Each mouse was placed in the central area of the maze, oriented towards one of the open arms. The SuperFcs animal behavior video analysis system (Model: XR-XC404, Shanghai Xinsoft Information Technology Co., Ltd.) was utilized to record the time spent and the number of entries into the open arms during a 5-minute session.

#### 2.3.3 Forced swim test

For the assessment of depression-like behavior, the forced swim test was conducted (70, 71)., modified from the method of Borsini (72). Mice were placed individually in a cylindrical glass container (12 cm in diameter, 30 cm in height) filled with water to a depth of 25 cm, maintained at a temperature of 24°C.The SuperFcs animal behavior video analysis system was again employed to monitor and record changes in the mice’s movements over a 6-minute period. The water depth was adjusted to prevent the animals from reaching the bottom of the container with their tails or hind limbs. The behavior during the last 4 minutes of the test was analyzed, with the duration of immobility during this period recorded using the SuperFcs animal behavior video analysis system (Model: XR-XC404, Shanghai Xinsoft Information Technology Co., Ltd.).

#### 2.3.4 Sugar water splash test

The splash test was conducted to evaluate depression-like behavior in the mice (73, 74). Each mouse was placed in an empty cage, and a 9% sucrose solution was sprayed once onto its back using a spray bottle, delivering a consistent volume of 0.7 mL per spray. The latency to the initial grooming behavior was noted, along with the grooming frequency, both observed over a 5-minute period post-spray.

### 2.4 Samples collection

At 21.2 months of age, fourteen mice were randomly selected from each of the control, model and QFY medium−dose groups (seven males and seven females per group). After ocular enucleation, blood was collected into anticoagulant EP tubes and kept on ice for 30min. Samples were then centrifuged at 3,500rpm for 15min at 4°C, and the resulting plasma supernatant was carefully harvested for downstream analyses.

### 2.5 Enzyme-linked immunosorbent assay

ELISA was utilized to quantify the levels of CORT, T, and E2 in the plasma samples of the mice. The ELISA kits were procured from Jiubang Biotechnology Co., Ltd. (CORT: Catalog No. ED-270, Batch No. 20240301; T: Catalog No. ED-244, Batch No. 20240301; E2: Catalog No. ED-251, Batch No. 20240325). The necessary strips were removed from the foil pouch after being equilibrated to room temperature for 60 minutes, and the remaining strips were sealed in a ziplock bag and stored at 4°C. Standard wells and sample wells were set up, with 50 μL of standards added to each standard well and 50 μL of diluted samples added to each sample well, no reagent was added to the blank wells. HRP-conjugated detection antibody (90 μL) were added to each standard and sample well (except for the blank wells). The wells were sealed with a plate cover and incubated at 37°C in a water bath or incubator for 60 minutes. After incubation, the liquid was discarded, and the wells were blotted dry on absorbent paper. Each well was filled with 350 μL of wash solution, left to stand for 1 minute, and then the wash solution was discarded, and the wells were blotted dry on absorbent paper. This washing process was repeated 5 times (a plate washer may also be used). Each well was then filled with 50 μL of substrate A and 50 μL of substrate B, and incubated at 37°C in the dark for 15 minutes. Finally, 50 μL of stop solution was added to each well, and the optical density (OD) at 450 nm was measured within 15 minutes. The OD values of the standards were plotted on the x-axis, and the corresponding concentrations on the y-axis, to create a standard curve using graph paper or appropriate software. The sample concentrations were then calculated by substituting the OD values of the samples into the four-parameter regression equation obtained from the standard curve.

### 2.6 Radioimmunoassay

Radioimmunoassay (RIA) was used to determine the concentrations of ACTH, FSH, LH, CRH, and GnRH in the plasma of the mice. The RIA kits were obtained from Beijing Furui Biotech Co., Ltd. (ACTH: Catalog No. RP-009, Batch No. 20240501; FSH: Catalog No. RK-176, Batch No. 20240525; LH: Catalog No. PK-117, Batch No. 20240501; CRH: Catalog No. RJ-063, Batch No. 20240501; GnRH: Catalog No. RP-6, Batch No. 20240501). All reagents were allowed to equilibrate to room temperature prior to utilization. Disposable 12 × 75 mm tubes were arranged in a test tube rack for the total count (TC) tube, non-specific binding (NSB) tube, zero standard (0) tube, standard tubes, and sample tubes. Fifty microliters of buffer were added to the NSB and 0 tubes, while 50 μL of the corresponding standards were added to the standard tubes. Fifty microliters of the corresponding samples were added to the sample tubes. One hundred microliters of antibody were added to all tubes except the NSB and 0 tubes. Then, 90 μL of 125I-ACTH radiolabeled tracer was added to all tubes. Each tube was vortexed for 9 seconds and then incubated in a 37°C water bath for 1 hour. After incubation, the tubes were removed, and 500 μL of PR separator was added to each tube. The tubes were vortexed thoroughly and left at room temperature for 20 minutes before being centrifuged at 3500 rpm at 4°C for 25 minutes. After centrifugation, the supernatant was discarded, and the precipitate in each tube was counted for radioactivity (cpm). The percentage of binding in the NSB and 0 tubes was calculated using B/T, and the percentage of binding in the standards and samples was calculated using B/Bo. A standard curve was plotted on semilogarithmic graph paper, or the gamma counter software was used to calculate the parameters, standard curve, and sample concentrations directly. The NSB% was calculated as (cpm of NSB tube ÷ cpm of T tube) × 90%, Bo% was calculated as (cpm of Bo tube - cpm of NSB tube) ÷ cpm of T tube × 90%, and B/Bo% was calculated as (cpm of standard or sample tube - cpm of NSB tube) ÷ (cpm of Bo tube - cpm of NSB tube) × 90%.

### 2.7 Statistical analysis

Data are presented as “Mean ± S.D.” The criterion for excluded data is that data outside 2 standard deviations are considered outliers. For statistical analysis, differences between two groups were assessed using Student’s *t*-test, while one-way Analysis of Variance (ANOVA) complemented by Dunnett’s test for multiple comparisons was applied to evaluate differences across three or more groups. The statistical analyses were performed using GraphPad Prism software version 10.4.1, and *P*-value of less than 0.05 was established as the threshold for statistical significance.

### 2.8 Principal component analysis

PCA is a statistical method that reduces a dataset with numerous variables to a smaller set of summary indices, preserving much of the information from the original variable set. These new values, referred to as principal components, are linear combinations of the original variables and are uncorrelated with one another. PCA facilitates the simplification of large data tables, making it easier to identify the most significant variables and to uncover patterns within the data. To differentiate between control, model, and QFY mice based on hormone levels in the HPA, HPT, and HPO axes. PCA was performed on the hormone levels and a score plot was generated. The PCA was conducted using GraphPad software.

### 2.9 Pearson correlation analysis and multiple linear regression analysis

To assess the linear relationship between variables, we employed Pearson correlation analysis, a widely used statistical method for quantifying the strength and direction of the association between two continuous variables. Pearson’s correlation coefficient (r) ranges from -1 to +1, where values closer to +1 or -1 indicate stronger positive or negative correlations, respectively, and a value of 0 suggests no correlation. Correlation significance was determined with p-values, where a threshold of p < 0.05 was considered statistically significant.

Multiple linear regression analysis is a statistical method used to modeling a linear relationships between a dependent variable (commonly denoted as Y) and multiple independent variables (usually represented as X1, X2,…, Xn). The magnitude of the regression coefficients reflects the extent of the effect each independent variable exerts on the dependent variable: larger values correspond to a greater influence, while smaller value indicate a lesser influence. Multiple linear regression analysis was performed using SPSS, and visualization was conducted with GraphPad software.

## 3 Results

### 3.1 The treatment of QFY ameliorated anxiety-like behavior in 3×Tg-AD mice

**Figure 1:**
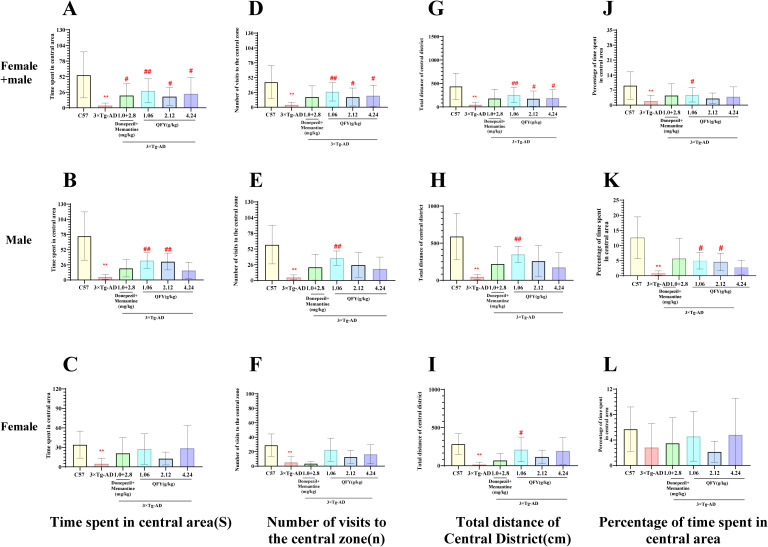
The effect of QFY on anxiety-like behavior of 3×Tg-AD transgenic mice in open field test. **(A-C)**, Time spent in central area; **(D–F)**, Number of visits to the central zone; **(G-I)**, Total distance in central district; **(J–L)**, Percentage of central area time. Mean ± S.D., n=7-20; ^**^ *P*<0.01 vs C57 mice, Student`s *t*-test; ^#^ *P*<0.05, ^##^ *P*<0.01 vs 3×Tg-AD mice, One-way ANOVA followed by Dunnett’ s multiple comparisons test; GraphPad 8.0.1.

The open field test results showed that 3×Tg-AD mice demonstrated a marked decrease in several parameters compared to wild-type mice, including the time spent in central area (**Figure 1A**, *P*<0.0001, B, *P*=0.0004, *F*=90.33, **Figure 1C**, *P*=0.0043), the number of visits to the central zone (**Figure 1D**, *P*<0.0001, E, *P*=0.0004, *F*=61.58, **Figure 1F**, *P*<0.01), total distance in central district (**Figure 1G**, *P*<0.0001, H, *P*=0.0003, *F*=80.56, I, *P*=0.0020), and the percentage of time spent in central area (**Figure 1J**, *P*<0.0001, K, *P*=0.0004, *F*=90.33).

In male mice, compared with the model group, the low-dose QFY group exhibited a significant increase in the time spent in central area (**Figure 1B**, *P*=0.2269), the number of visits to the central area (**Figure 1E**, *P*=0.0022), the total distance in central district (**Figure 1H**, *P*=0.0027) and the percentage of time spent in central area (**Figure 1K**, *P*=0.0123), medium-dose QFY group also demonstrated a significant increase in the time spent in central area (**Figure 1B**, *P*=0.0086) and the percentage of time spent in central area (**Figure 1K**, *P*=0.0439), no significant changes were observed in the high-dose group compared with the model group.

In female mice, compared with the model group, the low-dose QFY group exhibited a significant increase in the total distance in central district (**Figure 1I**, *P*=0.0402).

**Figure 2:**
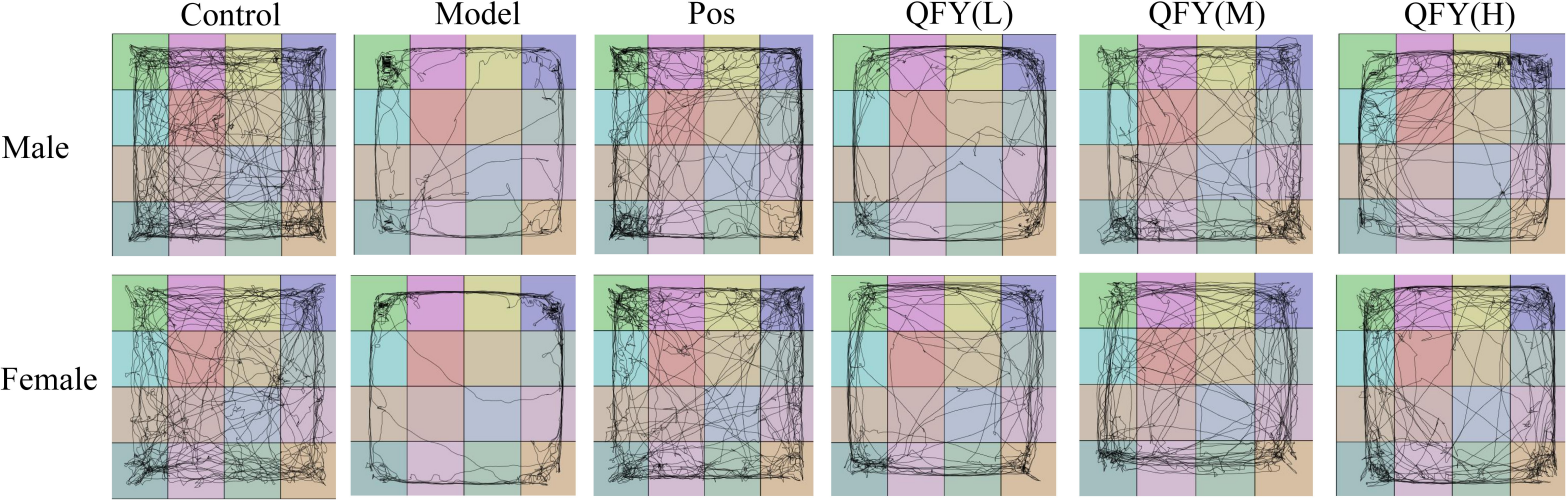
Open field test trajectory map of mice.

The combined analysis of both sexes showed that compared with the model group, the positive medicine group showed a significant increase in the time spent in central area. (**Figure 1A**, *P*=0.0387), the low-dose QFY group exhibited a significant increase in the time spent in central area (**Figure 1A**, *P*=0.0005), the number of visits to the central area (**Figure 1D**, *P*=0.0004), the total distance in central district (**Figure 1G**, *P*=0.0011) and the percentage of time spent in central area (**Figure 1J**, *P*=0.0146), the medium-dose and the high-dose QFY group also demonstrated a significant increase in the time spent in central area (**Figure 1A**, the medium-dose *P*=0.0213, the high-dose *P*=0.0186), the number of visits to the central area (**Figure 1D**, the medium-dose *P*=0.0339, the high-dose *P*=0.0209) and the total distance in central district (**Figure 1G**, the medium-dose *P*=0.0465, the high-dose *P*=0.0448). Furthermore, both male and female mice in the Model group exhibited a marked reduction in central area movement trajectories, which were significantly increased following treatment with the positive control medicine or QFY (**Figure 2**).

**Figure 3:**
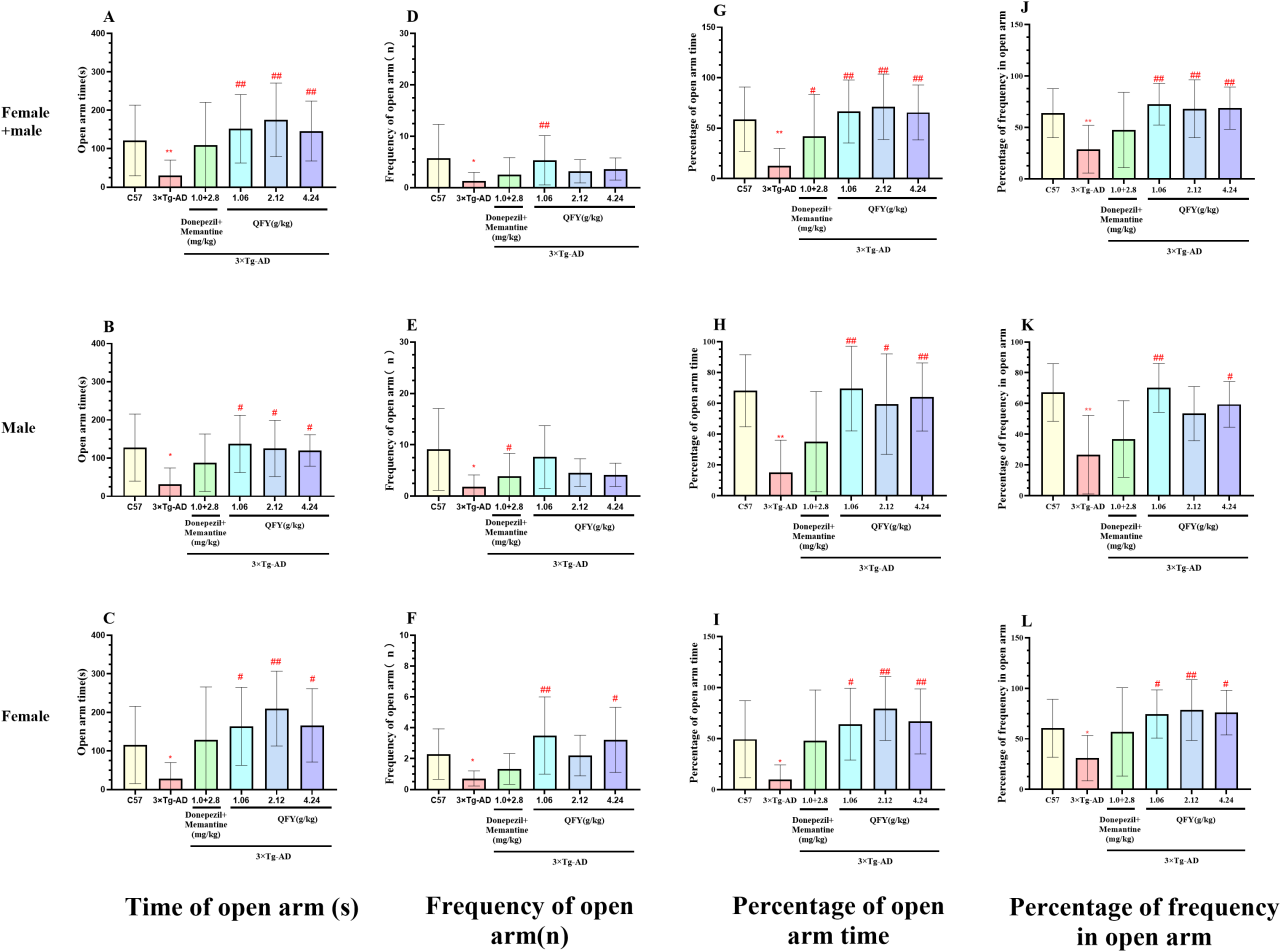
The effect of QFY on anxiety-like behavior of 3×Tg-AD transgenic mice in elevated plus maze test. **(A-C)**, Time of open arm; **(D-F)**, Frequency of open arm entry times; **(G-I)**, Percentage of open arm time; **(J-L)**, Percentage of frequency in open arm time. Mean ± S.D. n=7-9. ^*^ *P*<0.05, ^**^ *P*<0.01 vs C57 mice, Student`s *t*-test; ^#^ *P*<0.05, ^##^ *P*<0.01 vs 3×Tg-AD mice, One-way ANOVA followed by Dunnett’s multiple comparisons test, GraphPad 8.0.1.

In the elevated plus maze test, compared to wild-type mice, 3×Tg-AD mice showed a significant reduction in the time spent in open arm (**Figure 3A**, *P*=0.0002, B, *P*=0.0097, C, *P*=0.0133), the frequency of open arm (**Figure 3D**, *P*=0.0005, E, *P*=0.0067, F, *P*=0.0126), the percentage of time and frequency in open arm (**Figure 3G**, *P*<0.0001, H, *P*=0.0012, J, *P*=0.0005, K, *P*=0.0033, I, *P*=0.0030).

In male mice, compared with the model group, the QFY low-dose group exhibited a significant increase in both the time (**Figure 3B**, *P*=0.0157) and the frequency of open arms (**Figure 3E**, *P*=0.0138), the percentage of open arm time (**Figure 3H**, *P*=0.0052) and the percentage of frequency in open arms (**Figure 3K**, *P*=0.0021). The medium-dose and the high-dose group exhibited a significant increase the time spent in open arms (**Figure 3B**, the medium-dose *P*=0.0439, the high-dose *P*=0.0306) and the percentage of time spent in open arms (**Figure 3H**, the medium-dose *P*=0.0439, the high-dose *P*=0.0264).

In female mice, compared with the model group, the QFY low-dose group exhibited a significant increase the time spent in open arms (**Figure 3C**, *P*=0.0400), the frequency of open arm (**Figure 3F** *P*=0.0059) and the percentage of open arm time (**Figure 3I**, *P*=0.0307), the medium -dose group exhibited a significant increase the time spent in open arms (**Figure 3C**, *P*=0.0030), the percentage of time and frequency in open arm (**Figure 3I**, *P*=0.0018, L, *P*=0.0165), the high-dose group exhibited a significant increase in both the time (**Figure 3C**, *P*=0.0266) and the frequency of open arm (**Figure 3F**, *P*=0.0085), the percentage of open arm time (**Figure 3I**, *P*=0.0267) and the percentage of frequency in open arm (**Figure 3L**, *P*=0.0451).

The combined analysis of both sexes showed that compared with the model group, the QFY low-dose and the high-dose group exhibited a significant increase in both the time (**Figure 3A**, the low-dose *P*=0.0005, the high-dose *P*=0.0008) and the frequency of open arms (**Figure 3D**, the low-dose *P*=0.0002, the high-dose *P*=0.0052), the percentage of spent in open arm time (**Figure 3G**, the low-dose *P*=0.0002, the high-dose *P*=0.0005) and the percentage of frequency in open arms (**Figure 3J**, the low-dose *P*=0.0002, the high-dose *P*=0.0012), the medium-dose group exhibited a significant increase the time spent in open arms (**Figure 3A**, *P*<0.0001), the frequency of open arms (**Figure 3D**, *P*<0.05) with notable elevations in both the percentage of time spent in open arm (**Figure 3G**, *P*<0.0001) and the percentage of frequency in open arms (**Figure 3J**, *P*=0.0026).

These findings indicated that 3×Tg-AD mice exhibit anxiety-like symptoms, and that administration of QFY effectively mitigates these behaviors. Additionally, the combination therapy of donepezil and memantine did not have a significant impact on anxiety-like behavior in 3×Tg-AD mice.

### 3.2 The treatment of QFY alleviated depressive-like behavior in 3×Tg-AD mice

**Figure 4:**
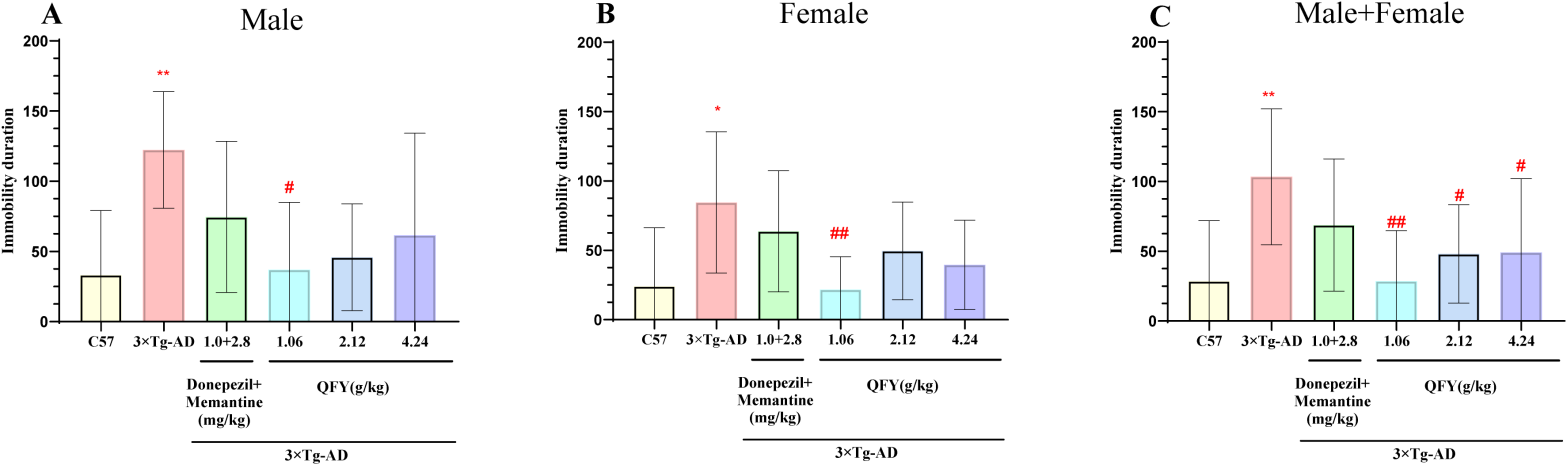
The effect of QFY on depressive-like behavior in 3×Tg-AD transgenic mice in the forced swimming experiment. **(A)** The immobility time of Male mice. **(B)** The immobility time of Female mice. **(C)** The immobility time of male + female mice. Mean ± S.D.n=7-9.^*^ *P*<0.05, ^**^ *P*<0.01 vs C57 mice, Student`s *t*-test; ^#^ *P*<0.05, ^##^ *P*<0.01 vs 3×Tg-AD mice, One-way ANOVA followed by Dunnett’s multiple comparisons test, GraphPad 8.0.1.

The outcomes of the forced swim test indicated that 3×Tg-AD mice had a notably longer immobility duration compared to their wild-type counterparts (**Figure 4A**, *P*=0.0019, B, *P*=0.0183, C, *P*<0.0001). In male mice, the low-dose QFY group exhibited a significant decrease in immobility duration compared to the model group (**Figure 4A**, *P*=0.0241). Similarly, in female mice, the low-dose QFY group showed a significant reduction in immobility duration compared to the model group (**Figure 4B**, *P*=0.0050, *F*=3.438). When analyzing both sexes combined, the QFY group demonstrated a significantly reduced immobility duration compared to the model group (**Figure 4C**, low-dose *P*=0.0001, medium-dose *P*=0.0232, high-dose *P*=0.0210).

**Figure 5:**
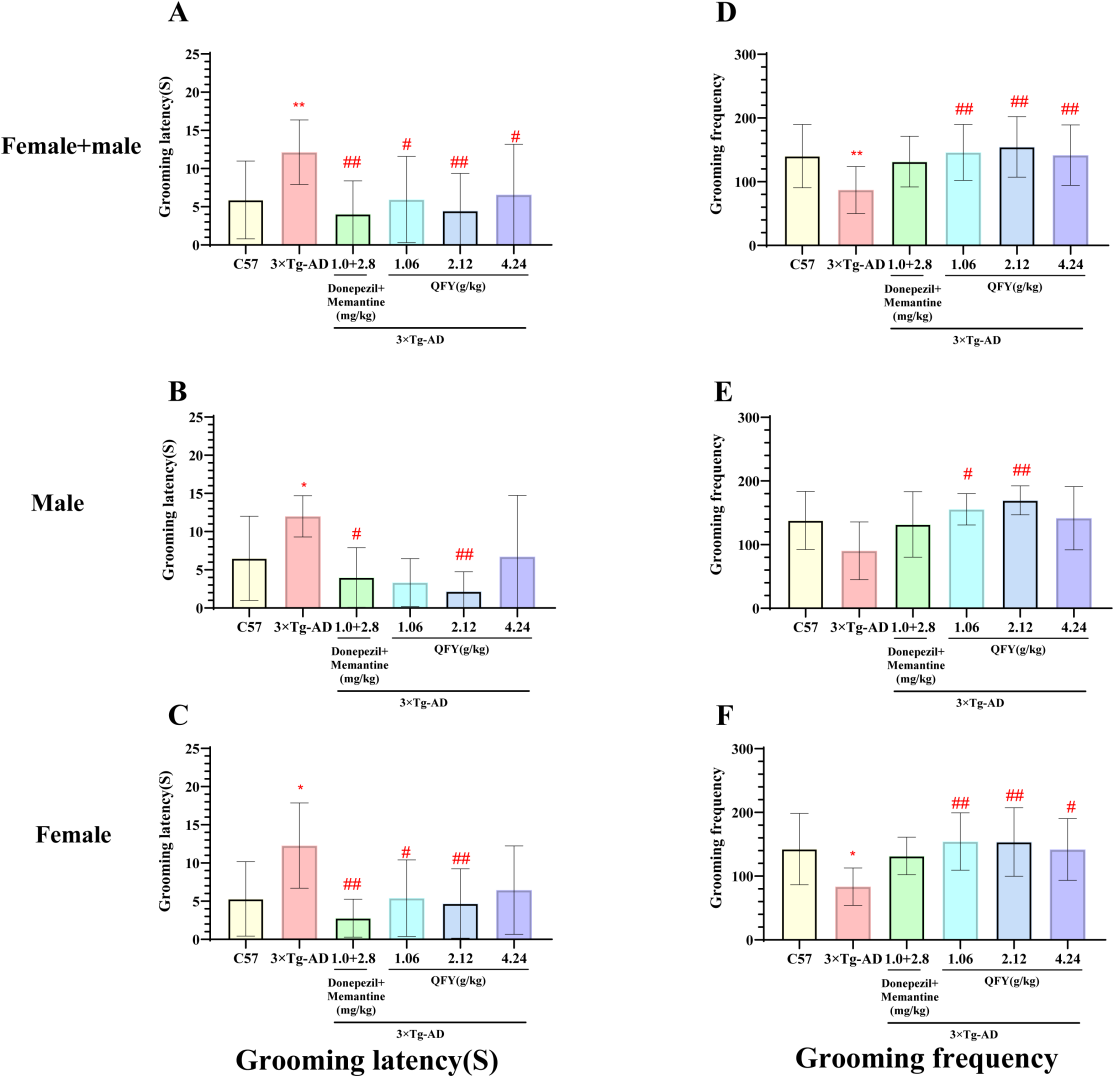
The effect of QFY on depressive-like behavior in 3×Tg-AD transgenic mice in the sugar water splash test. **(A-C)**, Grooming latency; **(D-F)**, Grooming frequency. Mean ± S.D., n=7-20; ^*^ *P*<0.05, ^**^ *P*<0.01 vs C57 mice, Student`s *t*-test; ^#^ *P*<0.05, ^##^ *P*<0.01 vs 3×Tg-AD mice, One-way ANOVA followed by Dunnett’s multiple comparisons test; GraphPad 8.0.1.

The results of sucrose splash test demonstrated that 3×Tg-AD mice exhibited a significantly longer grooming latency (**Figure 5A**, *P*=0.0006; B, *P*=0.0281, *F*=4.129, C, *P*=0.0152, *F*=1.313) and a notable decrease in grooming frequency (**Figure 5D**, *P*=0.0018, F, *P*=0.0225, *F*=3.617) compared to wild-type mice.

In male mice, compared to the model group, the positive medicine group showed a significant reduction in grooming latency (**Figure 5B**, *P*=0.0274). The QFY medium-dose group also exhibited a significant decrease in grooming latency (**Figure 5B**, *P*=0.0080) and a significant increase in grooming frequency (**Figure 5E**, *P*=0.0072, *F*=3.403). However, the QFY low-dose group showed a significant reduction in grooming frequency (**Figure 5E**, *P*=0.0317, *F*=3.403).

In female mice, compared to the model group, the positive medicine group demonstrated a significant shortening of grooming latency (**Figure 5C**, *P*=0.0012, *F*=4.275). The QFY groups also exhibited a significant reduction in grooming latency (**Figure 5C**, low-dose, *P*=0.0192, medium-dose, *P*=0.0089, *F*=4.275) and a significant increase in grooming frequency (**Figure 5F**; low-dose *P*=0.0068, medium-dose, *P*=0.0075; high-dose group, *P*=0.0357, *F*=3.484).

The combined analysis of both sexes showed that compared with the model group, the grooming latency was significantly shortened in the positive medicine group (**Figure 5A**, *P*=0.0005, *F*=1.822) and the QFY groups (**Figure 5A**, low-dose *P*=0.0150, medium-dose *P*=0.0011, high-dose, *P*=0.0231), The grooming frequency was significantly increased in the QFY groups (**Figure 5D**, low-dose *P*=0.0037, medium-dose *P*=0.0007, high-dose, *P*=0.0074).

### 3.3 The treatment of QFY regulated neuroendocrine function in 3×Tg-AD mice

We selected the control group, model group, and QFY clinical dose (medium dose group) for plasma hormone analysis to study the regulatory effects of QFY on the plasma levels of the HPA and HPG axes in 3×Tg-AD mice.

**Figure 6:**
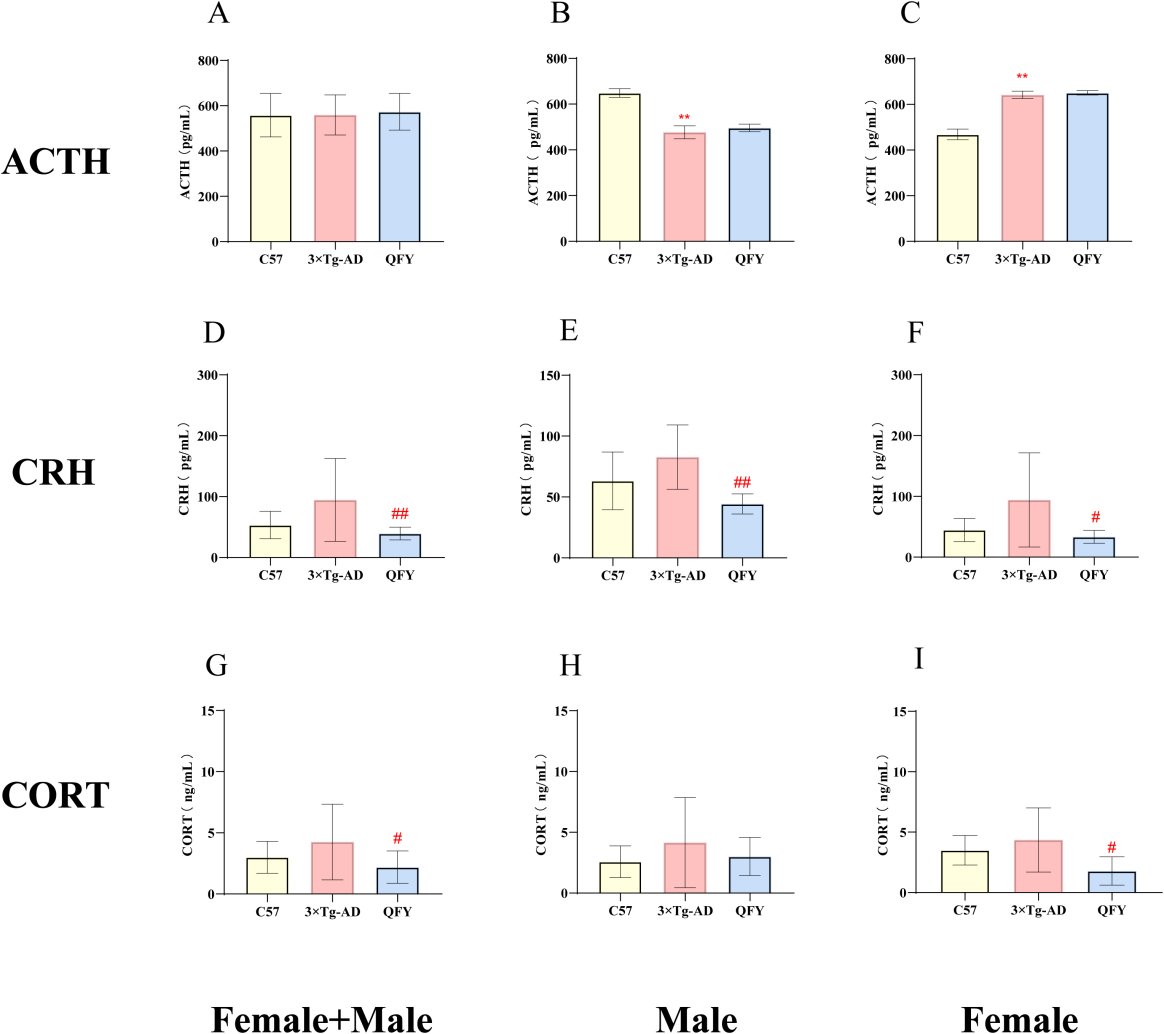
The effect of QFY on hypothalamic–pituitary–adrenal axis of 3×Tg-AD transgenic mice. **(A-C)** The concentration of ACTH in plasma. **(D-F)** The concentration of CRH in plasma. **(G-I)**, The concentration of CORT in plasma. Mean ± S.D., n=7-14. ^**^ *P*<0.01 vs C57 mice, Student`s *t*-test; ^#^ *P*<0.05, ^##^ *P*<0.01 vs 3×Tg-AD mice, Student`s *t*-test; Graphpad 8.0.1.

The results showed that, compared to wild-type mice, 3×Tg-AD mice had increased levels of ACTH in female (**Figure 6C**, *P*<0.0001, *F*=2.066) but decreased levels in males (**Figure 6B**, *P*<0.0001, *F*=2.198), CRH and CORT in the plasma. QFY group, a particularly notable reduction in CRH levels in both male and female mice (**Figure 6E**, *P*=0.0079, *F*=10.22, **Figure 6F**, *P*=0.0303) and CORT levels in female mice (**Figure 6I**, *P*=0.0111). These results indicate that 3×Tg-AD mice present hyperactive HPA axis, and the treatment of QFY balanced the HPA axis of 3×Tg-AD mice. The combined analysis of both sexes showed that plasma CRH and CORT levels in the QFY group were significantly lower than those in the model group (**Figure 6D**, *P*=0.0034, **Figure 6G**, *P*=0.0255).

**Figure 7:**
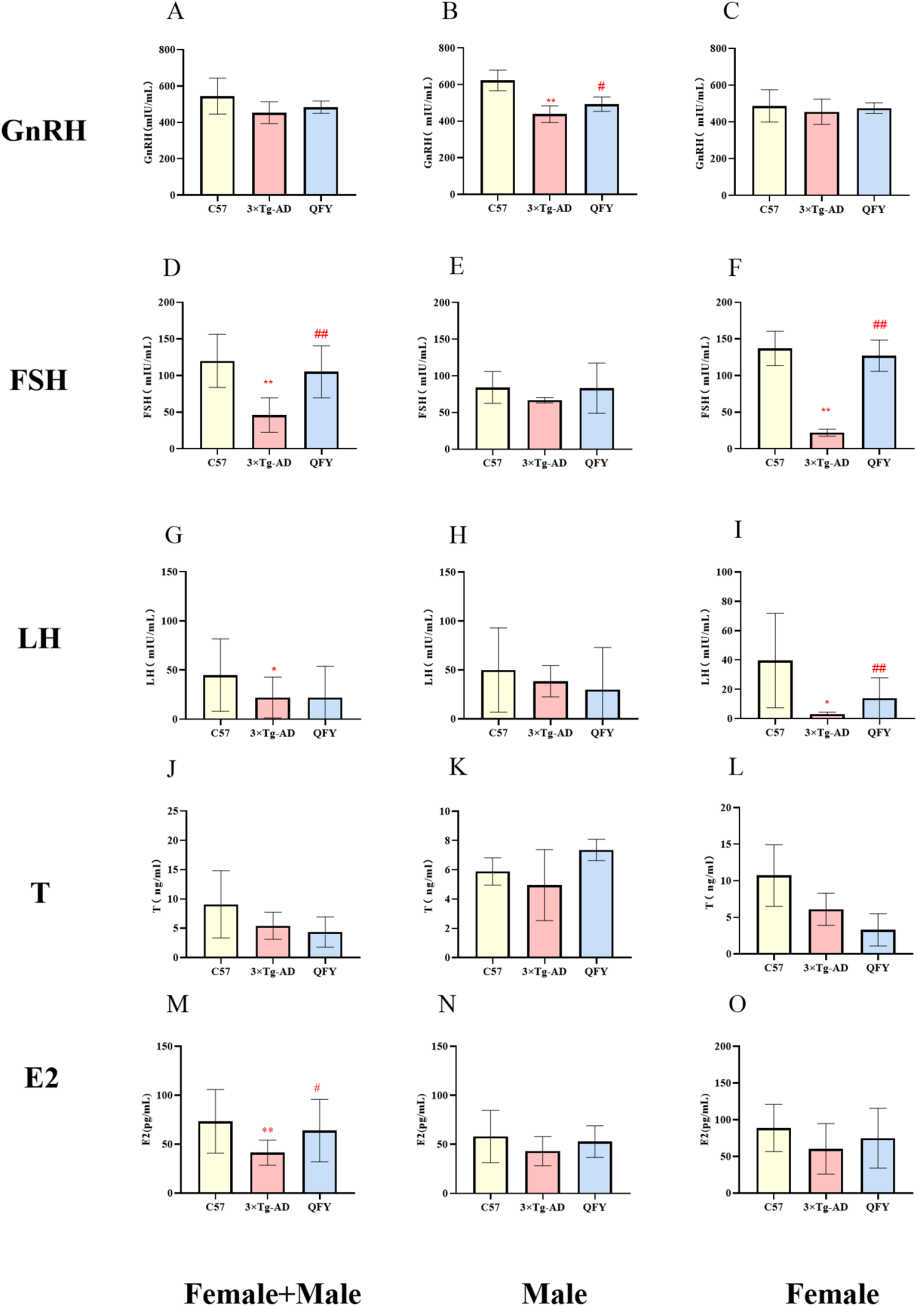
The effect of QFY on hypothalamic-pituitary-gonadal axis of 3×Tg-AD transgenic mice. **(A-C)** The concentration of GnRH in plasma. **(D-F)** The concentration of FSH in plasma. **(G-I)**, The concentration of LH in plasma. **(J-L)** The concentration of T in plasma. **(M-O)** The concentration of E2 in plasma. Mean ± S.D. n=7-14.^*^ *P*<0.05,^**^ *P*<0.01 vs C57 mice, Student`s *t*-test; ^#^ *P*<0.05, ^##^ *P*<0.01 vs 3×Tg-AD mice, Student`s *t*-test; Graphpad 8.0.1.

We measured the levels of GnRH, FSH, LH, T, and E2 in the plasma of mice. The results indicated that GnRH hormone levels decreased in male 3×Tg-AD mice (**Figure 7B**, *P*<0.0001, *F*=1.576) and the FSH, LH hormone levels decreased in female 3×Tg-AD mice (**Figure 7F**, *P*<0.0001, *F*=24.03, I, *P*=0.0188, *F*=730.3). The treatment with QFY reversed the low level of GnRH in male 3×Tg-AD mice (**Figure 7B**, *P*=0.0390, *F*=1.324) and the FSH (**Figure 7F**, *P*<0.0001, *F*=19.85), LH (**Figure 7I**, *P*=0.0012) in female 3×Tg-AD mice. These results suggest that the HPG axis was dysregulated in 3×Tg-AD mice, and the administration of QFY restored their balance. The combined analysis of both sexes showed that, compared with the control group, plasma FSH, LH, and E2 levels were significantly decreased in the model group (**Figure 7D**, *P*<0.0001 **Figure 7M**, *P*=0.0076, *F*=6.378, **Figure 7G**, *P*=0.0444), while plasma FSH and E2 levels were significantly increased in the QFY group (**Figure 7D**, *P*<0.0001, M, *P*=0.0476, *F*=6.137).

### 3.4 The specific neuroendocrine phenotype responding to the treatment of QFY

**Figure 8:**
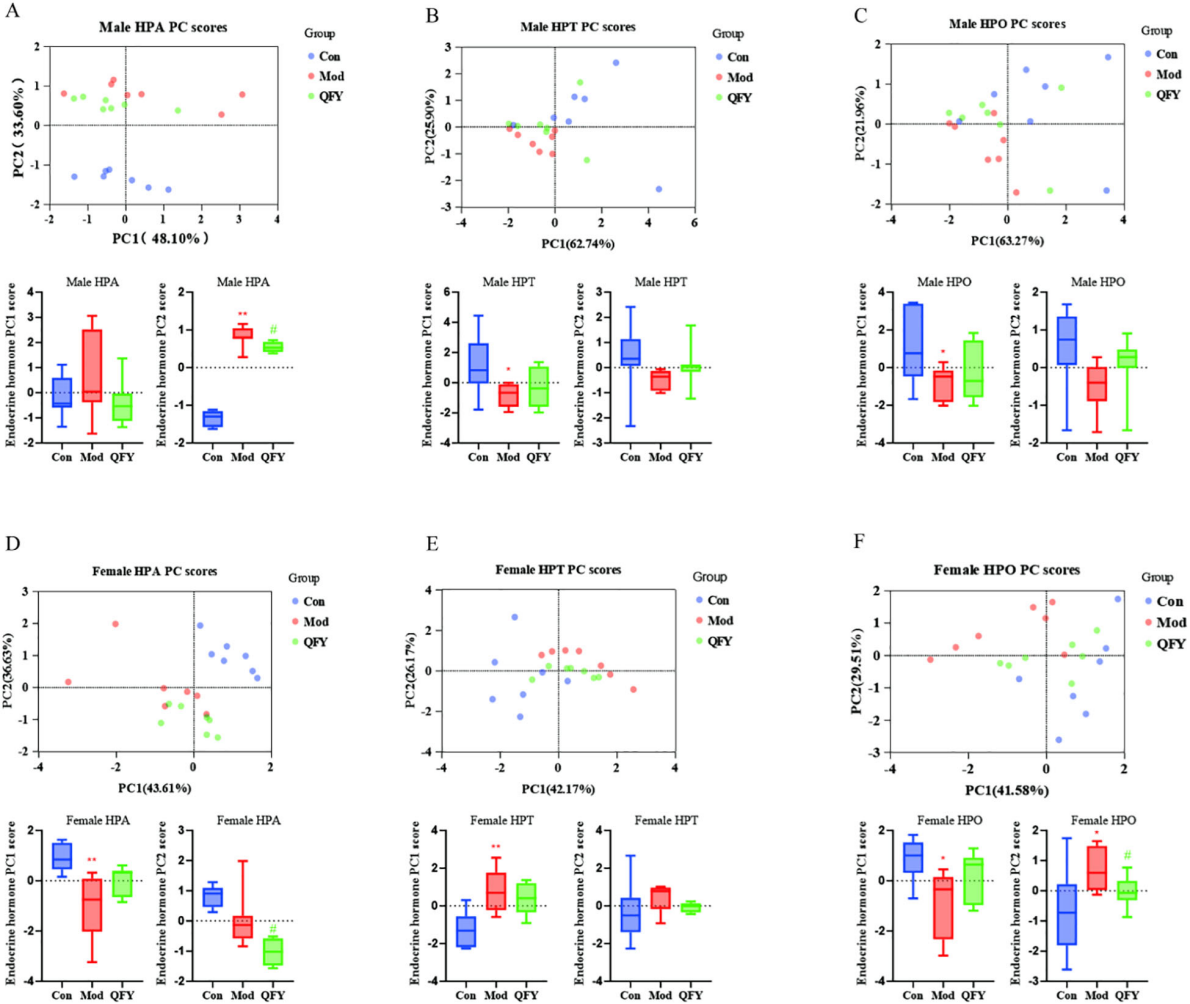
Principal component analysis based on the HPA, HPT, and HPO axes and the box plots of PCA score statistics. Each point represents a sample in PCA analysis plot: blue represents the Con group, red represents the Mod group, and green represents the QFY group, below each plot are box plots of PC1 and PC2 scores: Blue represents the Con group, red represents the Mod group, and green represents the QFY group. **(A-C)** the PCA analysis and box plots of PC1 and PC2 scores of the HPA, HPT, and HPO axes in male mice. **(D-F)** the PCA analysis and box plots of PC1 and PC2 scores of the HPA, HPT, and HPO axes in female mice. ^*^ *P*<0.05, ^**^ *P*<0.01 vs C57 mice, Student`s *t*-test; ^#^ *P*<0.05, vs 3×Tg-AD mice , Student`s *t*-test; Graphpad 8.0.1.

The principal component analysis (PCA) of HPA, HPT, and HPO among the control, model, and QFY groups in male mice was used to demonstrate a distinct differentiation between the control group and the combined model and QFY groups. To intuitively illustrate the spatial relationships among the three groups, we conducted a statistical analysis on the scores of the first two principal components, PC1 and PC2, derived from the PCA. The results showed that the male QFY group was positioned more proximate to the control group compared to the model group for the HPA PC scores (**Figure 8A**), and also the same that the female QFY group for the HPO PC scores (**Figure 8F**). The model group’s PC2 scores were significantly higher compared to those of the control group, and the QFY group’s scores were significantly reduced compared to those of model group for the HPA in male mice (**Figure 8A**) and HPO in female mice (**Figure 8F**). We observed that, for PC1, the scores of the model group were significantly lower compared to the control group (**Figure 8B, C, D, F**). However, for the HPT axis in female mice, the PC1 scores of the model group were significantly higher than those of the control group (**Figure 8E**). This indicated that HPA axis of male mice and HPO axis of female mice sensitively responded to the treatment of QFY.

### 3.5 The effects of QFY ameliorating anxiety and depression-like behaviors in 3×Tg-AD mice correlated with modulating the level of neuroendocrine hormones

We performed Pearson correlation analyses between hormones related to the HPA, HPT and HPO axes and the outcomes of the OFT, EPM, FST, and ST, and generating heatmaps to illustrate the relationship between BPSD and neuroendocrine function.

#### 3.5.1 The HPA axis

**Figure 9:**
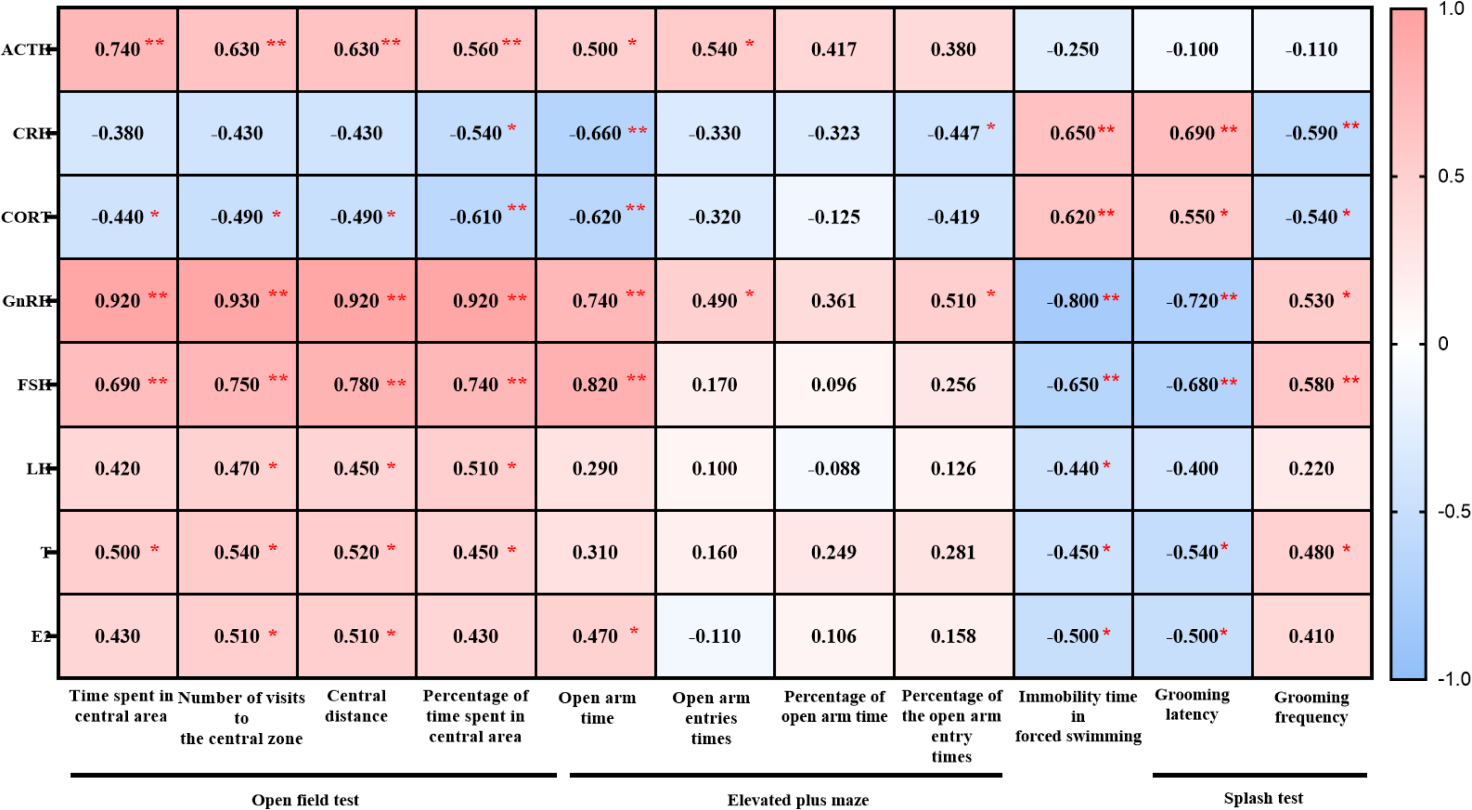
Heatmap generated from the correlation analysis between the open field test, elevated plus maze test, forced swimming, splash test and ACTH, CRH, CORT, GnRH, FSH, LH, T, E2 in male 3×Tg-AD mice. Colors closer to red indicate higher r-values, while colors closer to blue indicate lower r-values. ^*^ *P*<0.05, ^**^ *P*<0.01, The heatmap was created using GraphPad.

In male mice, plasma ACTH levels positively correlated with the indices of OFT (time in central zone, number of central zone entries, central distance, and percentage of central time). The levels of CRH and CORT negatively correlated with the percentage of frequency of open arm in the EPM but positively correlated with immobility time in the FST and grooming latency in the ST, while negatively correlated with grooming frequency (**Figure 9**).

**Figure 10:**
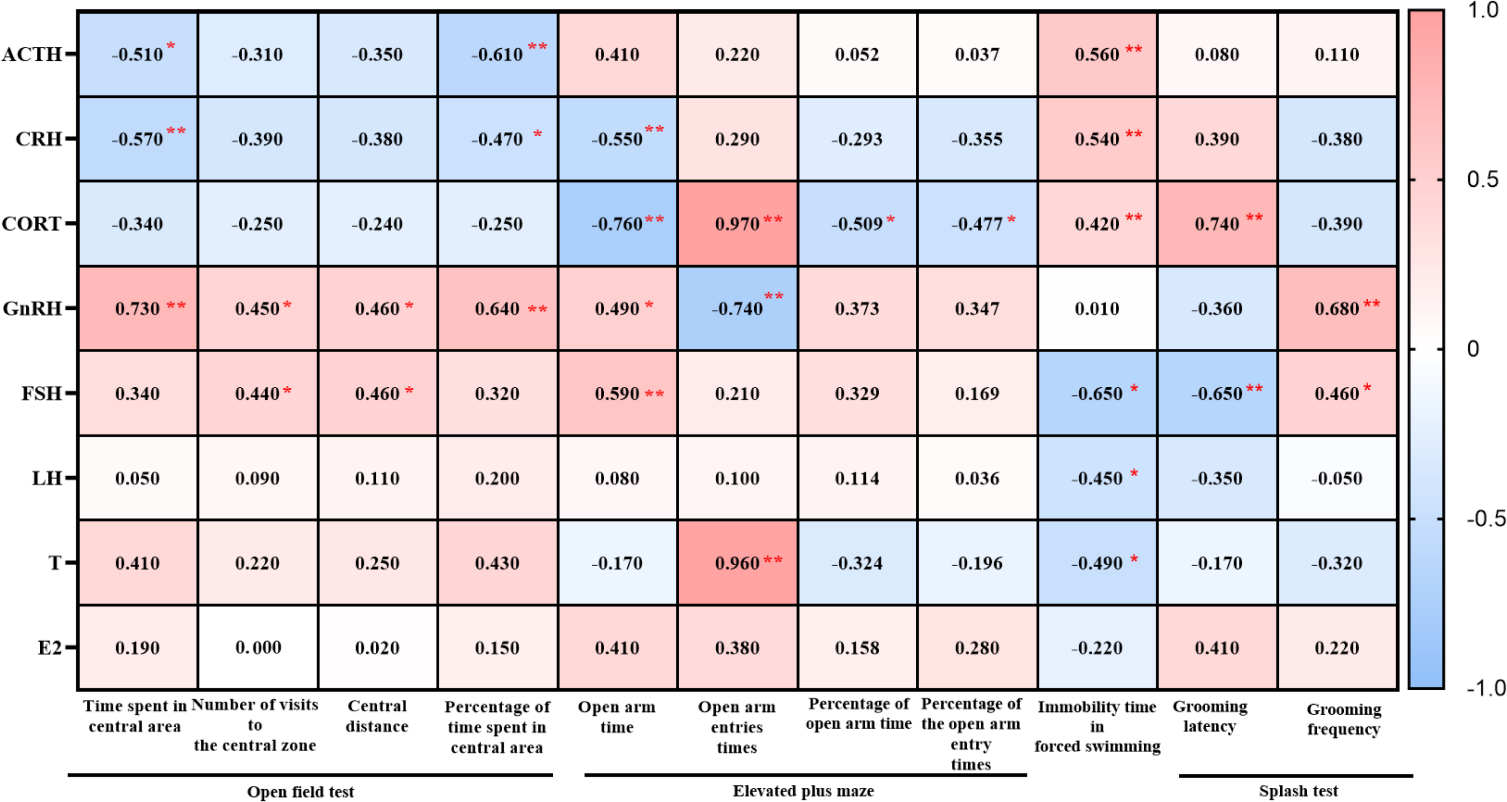
Heatmap generated from the correlation analysis between open field test, elevated plus maze test, forced swimming, splash test and ACTH, CRH, CORT, GnRH, FSH, LH, T, E2 in female 3×Tg-AD mice. Colors closer to red indicate higher r-values, while colors closer to blue indicate lower r-values. ^*^ *P*<0.05, ^**^ *P*<0.01, The heatmap was created using GraphPad.

In female mice, ACTH levels negatively correlated with central zone time and the percentage of central area time in the OFT but positively with immobility time in the FST. CRH levels positively correlated with immobility time in the FST and grooming latency in the ST. CORT levels negatively correlated with the open arm time in EPM, as well as the percentage of time and frequency in open arm time in EPM. but positively with the grooming latency in the ST (**Figure 10**).

#### 3.5.2 The HPT and HPO axes

In male mice, GnRH, FSH and T levels positively correlated with the indices of OFT (time in central zone, number of central zone entries, central distance, and percentage of central time). LH levels positively correlated with number of central zone entries, central distance, and percentage of central time in OFT. E2 levels positively correlated with number of central zone entries and central distance in OFT. As for EPM, GnRH, FSH, LH, T and E2 levels was positively with open arm entry times. GnRH was positively with the percentage of frequency in open arm. GnRH, FSH, LH, T and E2 was negatively correlated with the floating time in the FST. GnRH, FSH, T and E2 was negatively correlated with the grooming latency while GnRH, FSH, T positively with the grooming frequency in ST (**Figure 9**).

In female mice, GnRH was positively correlated with the indices of OFT (time in central zone, number of central zone entries, central distance, and percentage of central time), as well as the grooming frequency in ST, while negatively correlated with the grooming latency in ST. FSH was positively correlated with number of central zone entries, central distance in OFT and grooming frequency in ST, while negatively correlated with immobility time in the FST and grooming latency in the ST. LH and T levels was negatively correlated with immobility time in the FST (**Figure 10**).

### 3.6 Effects of endocrine hormones on anxiety and depression in mice

**Figure 11:**
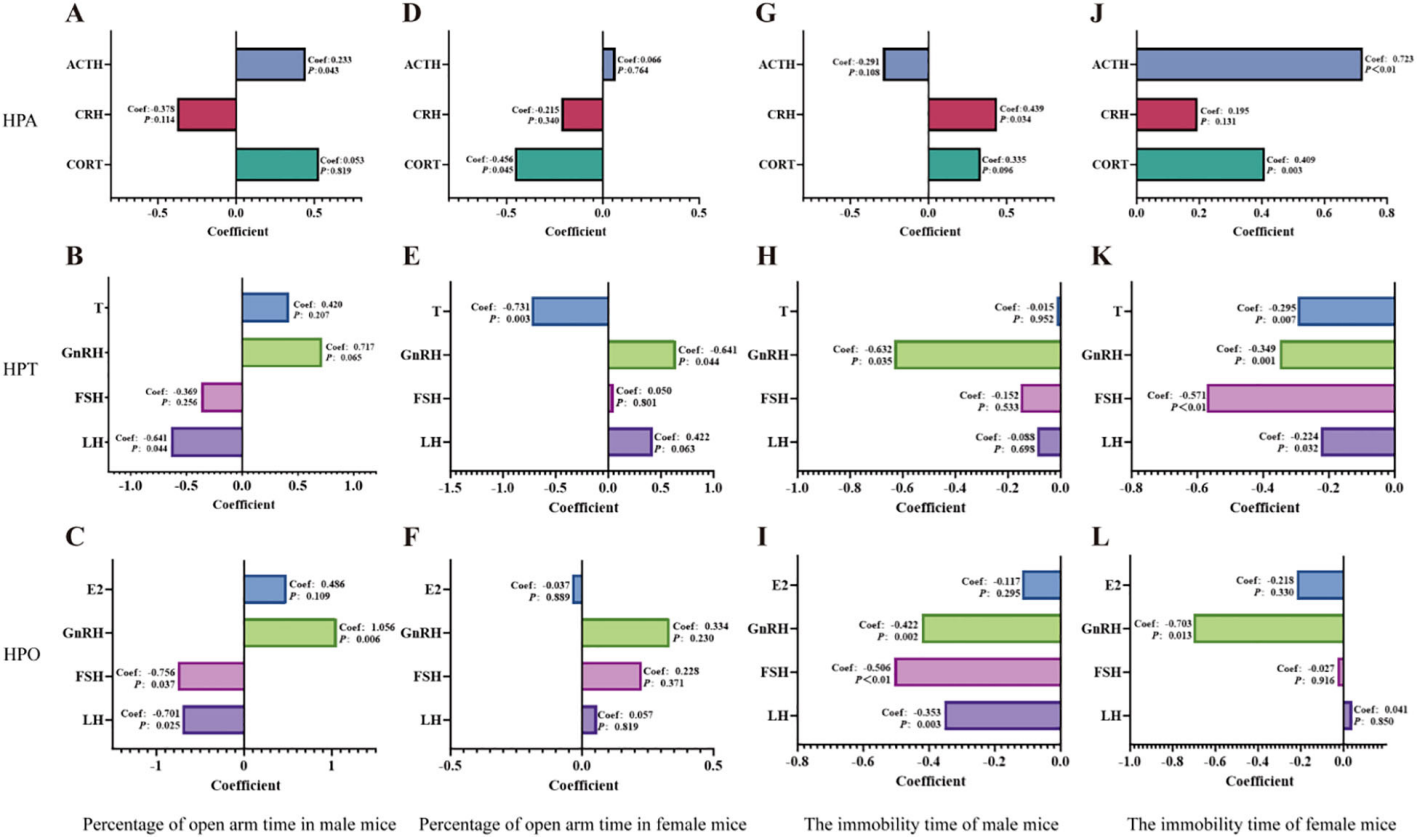
Multiple linear regression analyses of the HPA, HPT, and HPO axes were conducted to assess their relationships with the percentage of time spent in the open arms of the elevated plus maze and the duration of immobility in the forced swim test. **(A-C)**: Multiple linear regression analyses were conducted to the percentage of open-arm time in the EPM for male mice with the HPA, HPT and HPO axes. **(D-F)**: Multiple linear regression analyses were conducted to the percentage of open-arm time in the EPM for female mice with the HPA, HPT and HPO axes. **(G, H)**: Multiple linear regression analyses were conducted to immobility time in the FST for male mice with the HPA, HPT and HPO axes. **(J-L)**: Multiple linear regression analyses were conducted to examine immobility time in the FST for female mice across the HPA, HPT and HPO axes. Perform the calculations using IBM SPSS Statistics 27 and use Graphpad 8.0.1. for visualization.

The elevated plus maze and forced swim test are established methods for assessing anxiety-like and depression-like behaviors in animals respectively. We conducted multiple linear regression analyses to evaluate the contribution of HPA, HPT and HPO axes to the percentage of time spent in the open arms of the elevated plus maze, as well as immobility time in the forced swim test (**Figure 11**).

For the anxiety-like behaviors indicated by the percentage of time spent in the open arms of the elevated plus maze test, ACTH of HPA axis (**Figure 11A**), LH of HPT axis (**Figure 11B**) and GnRH of HPO axis (**Figure 11C**) (GnRH>LH>ACTH) dedicated to anxiety and respond to the treatment of QFY in male mice. While for female mice, it was CORT of HPA axis (**Figure 11D**), T of HPT axis (**Figure 11E**) (T>CORT).

For the depression -like behaviors indicated by immobility time in the forced swim test, the results showed that CRH of HPA axis (**Figure 11G**), GnRH of HPT and HPO axis (**Figure 11H, I**) (GnRH>CRH) dedicated to depression and respond to the treatment of QFY in male mice. While for female mice, it was ATCH of HPA axis (**Figure 11J**), FSH of HPT and HPO axes (**Figure 11K, L**) (ACTH>FSH).

These results indicated that GnRH was the most significant contributor to anxiety and depression in male 3×Tg-AD mice, while T and ACTH were major contributors to anxiety and depression in female 3×Tg-AD mice.

## 4 Discussion

The anxiety and depression-like symptoms are also present in certain animal models of AD. For instance, senescence-accelerated mice prone 8 (SAMP8) mice, which are utilized as a model for cognitive decline, have been observed to exhibit heightened aggression and anxiety-depression-like behaviors (22, 75). Similarly, the NL-GF and 5×FAD mouse models of AD have been noted to display behaviors indicative of anxiety and depression (76–78). Experimental analyses have demonstrated that 3×Tg-AD mice manifest BPSD (64), such as spending less time in the central area of the open field test (79) and exhibiting increased fecal boli (80), both of which are indicative of anxiety-like behaviors (81–83). In our study, we observed pronounced anxiety-like and depression-like symptoms in 3×Tg-AD mice. These mice not only spent less time in the central zone but also showed significant decreases in the number of visits to the central zone and the total distance traveled within it. Male mice, in particular, exhibited a notable reduction in the percentage of time spent in the central zone. Treatment with QFY significantly increased both the number of visits to the central zone and the total distance traveled, although it did not lead to a significant improvement in the time spent or percentage of time in that zone. These findings suggest that QFY alleviates anxiety symptoms primarily by enhancing exploratory behavior and activity levels. In the EPM, 3×Tg-AD mice exhibited significant reductions in open-arm time, the number of open-arm entries, the percentage of time spent in open-arm time, and the percentage of open-arm entries. Treatment with QFY significantly reversed these behaviors, indicating its potential to mitigate anxiety-like behaviors in 3×Tg-AD mice. We observed that 3×Tg-AD mice exhibited prolonged immobility time in the forced swim test, which is consistent with previous research findings (84), the immobility time in the QFY treatment group was significantly reduced. Our study reported that 3×Tg-AD mice exhibited an extended grooming latency in the sucrose splash test, and in female mice, the grooming frequency was significantly reduced. In the QFY treatment group, grooming latency was significantly shortened, and grooming frequency was significantly increased. These findings suggest that QFY can improve depression-like behaviors in mice.

Some research has indicated that the HPA axis is hyperactivated in 3×Tg-AD mice (85, 86), which may contribute to the observed neuropsychiatric symptoms. This hyperactivation is primarily indicated by elevated CORT levels. At the genetic level, increased expression of CRH mRNA has been observed in specific brain regions (87).Generally, CRH stimulates downstream ACTH secretion, which in turn promotes adrenal secretion of CORT (88). In this study, we observed a trend towards increased CRH and CORT levels in 3×Tg-AD mice compared to wild-type. However, these differences were not statistically significant. One possible explanation for this lack of statistical significance is the high biological variability among samples in the 3×Tg-AD model, which may obscure subtle changes in CRH and CORT levels. Regarding ACTH levels, prior studies have shown that female rodents release more ACTH than males following stress exposure (89). We observed a similar phenomenon in 3×Tg-AD mice, where female mice exhibited higher ACTH levels than their male counterparts. To date, no studies have systematically investigated the HPT and HPO axes in 3×Tg-AD mice. In our present study, we found that T and E2 levels were partially suppressed in 3×Tg-AD mice. Interestingly, we found that testosterone levels in female 3×Tg-AD mice were higher than those in males. This finding is counterintuitive but may be explained by age-related hormonal alterations specific to this AD mouse model. Previous studies have reported that testosterone levels in male 3×Tg-AD mice decline dramatically with age (90, 91), there are also relevant reports in humans (92). In contrast, no consistent reports have described a similar decline in testosterone levels in female 3×Tg-AD mice. Therefore, we speculate that the higher T levels observed in females may not be due to an actual increase in androgen production but rather reflect a sharp age-related drop in circulating testosterone in male 3×Tg-AD mice. GnRH, a reproductive hormone secreted by the hypothalamus (93), plays a crucial role in the mammalian HPT and HPO axes by stimulating pituitary secretion of LH (94) and FSH (95, 96). Our findings revealed significant suppression of upstream components of the HPG axis in 3×Tg-AD mice. Specifically, male mice exhibited significantly reduced GnRH and FSH levels, while female mice showed significant reductions in FSH and LH levels. These results indicate that the upstream HPG axis is inhibited in 3×Tg-AD mice. Importantly, treatment with the QFY effectively reversed these alterations, demonstrating its regulatory potential on the HPG axis.

Previous researches have demonstrated that QFY improves cognitive function in AD models through mechanisms such as activating the Keap1/Nrf2/ARE signaling pathway (97), inhibiting the RAGE/NF-κB pathway (98), modulating gut microbiota (62), and regulating immune responses (63). However, no studies have specifically investigated the effects of QFY on BPSD associated with AD. In our study, we found that QFY alleviated symptoms of anxiety and depression in 3×Tg-AD mice by regulating the HPA, HPT and HPO axes.

There are also limitations in our study. The primary objective of this study was to evaluate the overall therapeutic effects of QFY on anxiety- and depression-like symptoms through modulation of the HPA and HPG axes. The current experimental design includes pre- and post-treatment assessments of plasma hormone levels and behavioral outcomes, which are sufficient to verify the efficacy of QFY. However, this design does not establish whether hormonal changes precede behavioral improvements. Given that endocrine fluctuations may serve as early indicators of behavioral changes in neuropsychiatric disorders, longitudinal monitoring of hormone levels at multiple time points (prior to, during, and after treatment) would provide critical insights into the temporal dynamics of QFY’s effects. Future studies should incorporate a time-course analysis to elucidate whether QFY-induced hormonal modulation occurs as a precursor to, or in parallel with, behavioral amelioration. Such an approach would further refine our understanding of the mechanistic pathways underlying QFY’s therapeutic efficacy and enhance its translational potential.

## Data Availability

The raw data supporting the conclusions of this article will be made available by the authors, without undue reservation.
