# Supplementary material for: Qifuyin alleviates anxiety and depression in 3×Tg-AD mice by modulating neuroendocrine function
Source: Front Psychiatry. 2025 May 14;16:1554866. doi: 10.3389/fpsyt.2025.1554866 (PMC12116680; doi:10.3389/fpsyt.2025.1554866)
Supplement: Supplementary file 1 [file DataSheet1.zip › Raw data/figure of individual data points/Pearson Scatter diagram of EPM.docx]

A, D:Scatterplot of correlation between male + female ACTH and Open arm time, Open arm entry times.

B, E:Scatterplot of correlation between male ACTH and Open arm time, Open arm entry times.

C, F:Scatterplot of correlation between female ACTH and Open arm time, Open arm entry times.

A, D:Scatterplot of correlation between male + female ACTH and percentage of open arm time, percentage of open arm entry times.

B, E:Scatterplot of correlation between male ACTH and percentage of open arm time, percentage of open arm entry times.

C, F:Scatterplot of correlation between female ACTH and percentage of open arm time, percentage of open arm entry times.

A, D:Scatterplot of correlation between male + female CRH and Open arm time, Open arm entry times.

B, E:Scatterplot of correlation between male CRH and Open arm time, Open arm entry times.

C, F:Scatterplot of correlation between female CRH and Open arm time, Open arm entry times.

A, D:Scatterplot of correlation between male + female CRH and percentage of open arm time, percentage of open arm entry times.

B, E:Scatterplot of correlation between male CRH and percentage of open arm time, percentage of open arm entry times.

C, F:Scatterplot of correlation between female CRH and percentage of open arm time, percentage of open arm entry times.

A, D:Scatterplot of correlation between male + female CORT and Open arm time, Open arm entry times.

B, E:Scatterplot of correlation between male CORT and Open arm time, Open arm entry times.

C, F:Scatterplot of correlation between female CORT and Open arm time, Open arm entry times.

A, D:Scatterplot of correlation between male + female CORT and percentage of open arm time, percentage of open arm entry times.

B, E:Scatterplot of correlation between male CORT and percentage of open arm time, percentage of open arm entry times.

C, F:Scatterplot of correlation between female CORT and percentage of open arm time, percentage of open arm entry times.

A, D:Scatterplot of correlation between male + female GnRH and Open arm time, Open arm entry times.

B, E:Scatterplot of correlation between male GnRH and Open arm time, Open arm entry times.

C, F:Scatterplot of correlation between female GnRH and Open arm time, Open arm entry times.

A, D:Scatterplot of correlation between male + female GnRH and percentage of open arm time, percentage of open arm entry times.

B, E:Scatterplot of correlation between male GnRH and percentage of open arm time, percentage of open arm entry times.

C, F:Scatterplot of correlation between female GnRH and percentage of open arm time, percentage of open arm entry times.

A, D:Scatterplot of correlation between male + female FSH and Open arm time, Open arm entry times.

B, E:Scatterplot of correlation between male FSH and Open arm time, Open arm entry times.

C, F:Scatterplot of correlation between female FSH and Open arm time, Open arm entry times.

A, D:Scatterplot of correlation between male + female FSH and percentage of open arm time, percentage of open arm entry times.

B, E:Scatterplot of correlation between male FSH and percentage of open arm time, percentage of open arm entry times.

C, F:Scatterplot of correlation between female FSH and percentage of open arm time, percentage of open arm entry times.

A, D:Scatterplot of correlation between male + female LH and Open arm time, Open arm entry times.

B, E:Scatterplot of correlation between male LH and Open arm time, Open arm entry times.

C, F:Scatterplot of correlation between female LH and Open arm time, Open arm entry times.

A, D:Scatterplot of correlation between male + female LH and percentage of open arm time, percentage of open arm entry times.

B, E:Scatterplot of correlation between male LH and percentage of open arm time, percentage of open arm entry times.

C, F:Scatterplot of correlation between female LH and percentage of open arm time, percentage of open arm entry times.

A, D:Scatterplot of correlation between male + female T and Open arm time, Open arm entry times.

B, E:Scatterplot of correlation between male T and Open arm time, Open arm entry times.

C, F:Scatterplot of correlation between female T and Open arm time, Open arm entry times.

A, D:Scatterplot of correlation between male + female T and percentage of open arm time, percentage of open arm entry times.

B, E:Scatterplot of correlation between male T and percentage of open arm time, percentage of open arm entry times.

C, F:Scatterplot of correlation between female T and percentage of open arm time, percentage of open arm entry times.

A, D:Scatterplot of correlation between male + female E2 and Open arm time, Open arm entry times.

B, E:Scatterplot of correlation between male E2 and Open arm time, Open arm entry times.

C, F:Scatterplot of correlation between female E2 and Open arm time, Open arm entry times.

A, D:Scatterplot of correlation between male + female E2 and percentage of open arm time, percentage of open arm entry times.

B, E:Scatterplot of correlation between male E2 and percentage of open arm time, percentage of open arm entry times.

C, F:Scatterplot of correlation between female E2 and percentage of open arm time, percentage of open arm entry times.
