# Supplementary material for: Qifuyin alleviates anxiety and depression in 3×Tg-AD mice by modulating neuroendocrine function
Source: Front Psychiatry. 2025 May 14;16:1554866. doi: 10.3389/fpsyt.2025.1554866 (PMC12116680; doi:10.3389/fpsyt.2025.1554866)
Supplement: Supplementary file 1 [file DataSheet1.zip › Raw data/figure of individual data points/Pearson Scatter diagram of FST.docx]

A:Scatterplot of correlation between male + female ACTH and floating time.

B:Scatterplot of correlation between male ACTH and floating time.

C:Scatterplot of correlation between female ACTH and floating time.

A:Scatterplot of correlation between male + female CRH and floating time.

B:Scatterplot of correlation between male CRH and floating time.

C:Scatterplot of correlation between female CRH and floating time.

A:Scatterplot of correlation between male + female CORT and floating time.

B:Scatterplot of correlation between male CORT and floating time.

C:Scatterplot of correlation between female CORT and floating time.

A:Scatterplot of correlation between male + female GnRH and floating time.

B:Scatterplot of correlation between male GnRH and floating time.

C:Scatterplot of correlation between female GnRH and floating time.

A:Scatterplot of correlation between male + female FSH and floating time.

B:Scatterplot of correlation between male FSH and floating time.

C:Scatterplot of correlation between female FSH and floating time.

A:Scatterplot of correlation between male + female LH and floating time.

B:Scatterplot of correlation between male LH and floating time.

C:Scatterplot of correlation between female LH and floating time.

A:Scatterplot of correlation between male + female T and floating time.

B:Scatterplot of correlation between male T and floating time.

C:Scatterplot of correlation between female T and floating time.

A:Scatterplot of correlation between male + female E2 and floating time.

B:Scatterplot of correlation between male E2 and floating time.

C:Scatterplot of correlation between female E2 and floating time.
