# Supplementary material for: Qifuyin alleviates anxiety and depression in 3×Tg-AD mice by modulating neuroendocrine function
Source: Front Psychiatry. 2025 May 14;16:1554866. doi: 10.3389/fpsyt.2025.1554866 (PMC12116680; doi:10.3389/fpsyt.2025.1554866)
Supplement: Supplementary file 1 [file DataSheet1.zip › Raw data/figure of individual data points/Pearson Scatter diagram of OFT.docx]

A, D, G, J: Scatterplot of correlation between male + female ACTH and central area time, Number of visits to the central zone, central distance, and percentage of central area time.

B, E, H, K: Scatterplot of correlation between male ACTH and central area time, Number of visits to the central zone, central distance, and percentage of central area time.

C, F, I, L:Scatterplot of correlation between female ACTH and central area time, Number of visits to the central zone, central distance, and percentage of central area time.

A, D, G, J: Scatterplot of correlation between male + female CRH and central area time, Number of visits to the central zone, central distance, and percentage of central area time.

B, E, H, K: Scatterplot of correlation between male CRH and central area time, Number of visits to the central zone, central distance, and percentage of central area time.

C, F, I, L:Scatterplot of correlation between female CRH and central area time, Number of visits to the central zone, central distance, and percentage of central area time.

A, D, G, J: Scatterplot of correlation between male + female CORT and central area time, Number of visits to the central zone, central distance, and percentage of central area time.

B, E, H, K: Scatterplot of correlation between male CORT and central area time, Number of visits to the central zone, central distance, and percentage of central area time.

C, F, I, L:Scatterplot of correlation between female CORT and central area time, Number of visits to the central zone, central distance, and percentage of central area time.

A, D, G, J: Scatterplot of correlation between male + female GnRH and central area time, Number of visits to the central zone, central distance, and percentage of central area time.

B, E, H, K: Scatterplot of correlation between male GnRH and central area time, Number of visits to the central zone, central distance, and percentage of central area time.

C, F, I, L:Scatterplot of correlation between female GnRH and central area time, Number of visits to the central zone, central distance, and percentage of central area time.

A, D, G, J: Scatterplot of correlation between male + female FSH and central area time, Number of visits to the central zone, central distance, and percentage of central area time.

B, E, H, K: Scatterplot of correlation between male FSH and central area time, Number of visits to the central zone, central distance, and percentage of central area time.

C, F, I, L:Scatterplot of correlation between female FSH and central area time, Number of visits to the central zone, central distance, and percentage of central area time.

A, D, G, J: Scatterplot of correlation between male + female LH and central area time, Number of visits to the central zone, central distance, and percentage of central area time.

B, E, H, K: Scatterplot of correlation between male LH and central area time, Number of visits to the central zone, central distance, and percentage of central area time.

C, F, I, L:Scatterplot of correlation between female LH and central area time, Number of visits to the central zone, central distance, and percentage of central area time.

A, D, G, J: Scatterplot of correlation between male + female T and central area time, Number of visits to the central zone, central distance, and percentage of central area time.

B, E, H, K: Scatterplot of correlation between male T and central area time, Number of visits to the central zone, central distance, and percentage of central area time.

C, F, I, L:Scatterplot of correlation between female T and central area time, Number of visits to the central zone, central distance, and percentage of central area time.

A, D, G, J: Scatterplot of correlation between male + female E2 and central area time, Number of visits to the central zone, central distance, and percentage of central area time.

B, E, H, K: Scatterplot of correlation between male E2 and central area time, Number of visits to the central zone, central distance, and percentage of central area time.

C, F, I, L:Scatterplot of correlation between female E2 and central area time, Number of visits to the central zone, central distance, and percentage of central area time.
