# Supplementary material for: Qifuyin alleviates anxiety and depression in 3×Tg-AD mice by modulating neuroendocrine function
Source: Front Psychiatry. 2025 May 14;16:1554866. doi: 10.3389/fpsyt.2025.1554866 (PMC12116680; doi:10.3389/fpsyt.2025.1554866)
Supplement: Supplementary file 1 [file DataSheet1.zip › Raw data/figure of individual data points/Pearson Scatter diagram of ST.docx]

A, D: Scatterplot of correlation between male + female ACTH and grooming latency, Grooming frequency.

B, E: Scatterplot of correlation between male ACTH and grooming latency, Grooming frequency.

C, F:Scatterplot of correlation between female ACTH and grooming latency, Grooming frequency.

A, D: Scatterplot of correlation between male + female CRH and grooming latency, Grooming frequency.

B, E: Scatterplot of correlation between male CRH and grooming latency, Grooming frequency.

C, F:Scatterplot of correlation between female CRH and grooming latency, Grooming frequency.

A, D: Scatterplot of correlation between male + female CORT and grooming latency, Grooming frequency.

B, E: Scatterplot of correlation between male CORT and grooming latency, Grooming frequency.

C, F:Scatterplot of correlation between female CORT and grooming latency, Grooming frequency.

A, D: Scatterplot of correlation between male + female GnRH and grooming latency, Grooming frequency.

B, E: Scatterplot of correlation between male GnRH and grooming latency, Grooming frequency.

C, F:Scatterplot of correlation between female GnRH and grooming latency, Grooming frequency.

A, D: Scatterplot of correlation between male + female FSH and grooming latency, Grooming frequency.

B, E: Scatterplot of correlation between male FSH and grooming latency, Grooming frequency.

C, F:Scatterplot of correlation between female FSH and grooming latency, Grooming frequency.

A, D: Scatterplot of correlation between male + female LH and grooming latency, Grooming frequency.

B, E: Scatterplot of correlation between male LH and grooming latency, Grooming frequency.

C, F:Scatterplot of correlation between female LH and grooming latency, Grooming frequency.

A, D: Scatterplot of correlation between male + female T and grooming latency, Grooming frequency.

B, E: Scatterplot of correlation between male T and grooming latency, Grooming frequency.

C, F:Scatterplot of correlation between female T and grooming latency, Grooming frequency.

A, D: Scatterplot of correlation between male + female E2 and grooming latency, Grooming frequency.

B, E: Scatterplot of correlation between male E2 and grooming latency, Grooming frequency.

C, F:Scatterplot of correlation between female E2 and grooming latency, Grooming frequency.
