# Supplementary material for: Qifuyin alleviates anxiety and depression in 3×Tg-AD mice by modulating neuroendocrine function
Source: Front Psychiatry. 2025 May 14;16:1554866. doi: 10.3389/fpsyt.2025.1554866 (PMC12116680; doi:10.3389/fpsyt.2025.1554866)
Supplement: Supplementary file 1 [file DataSheet1.zip › Raw data/figure of individual data points/Scatter plot of behavioral experiments and measurement of hormone levels.docx]

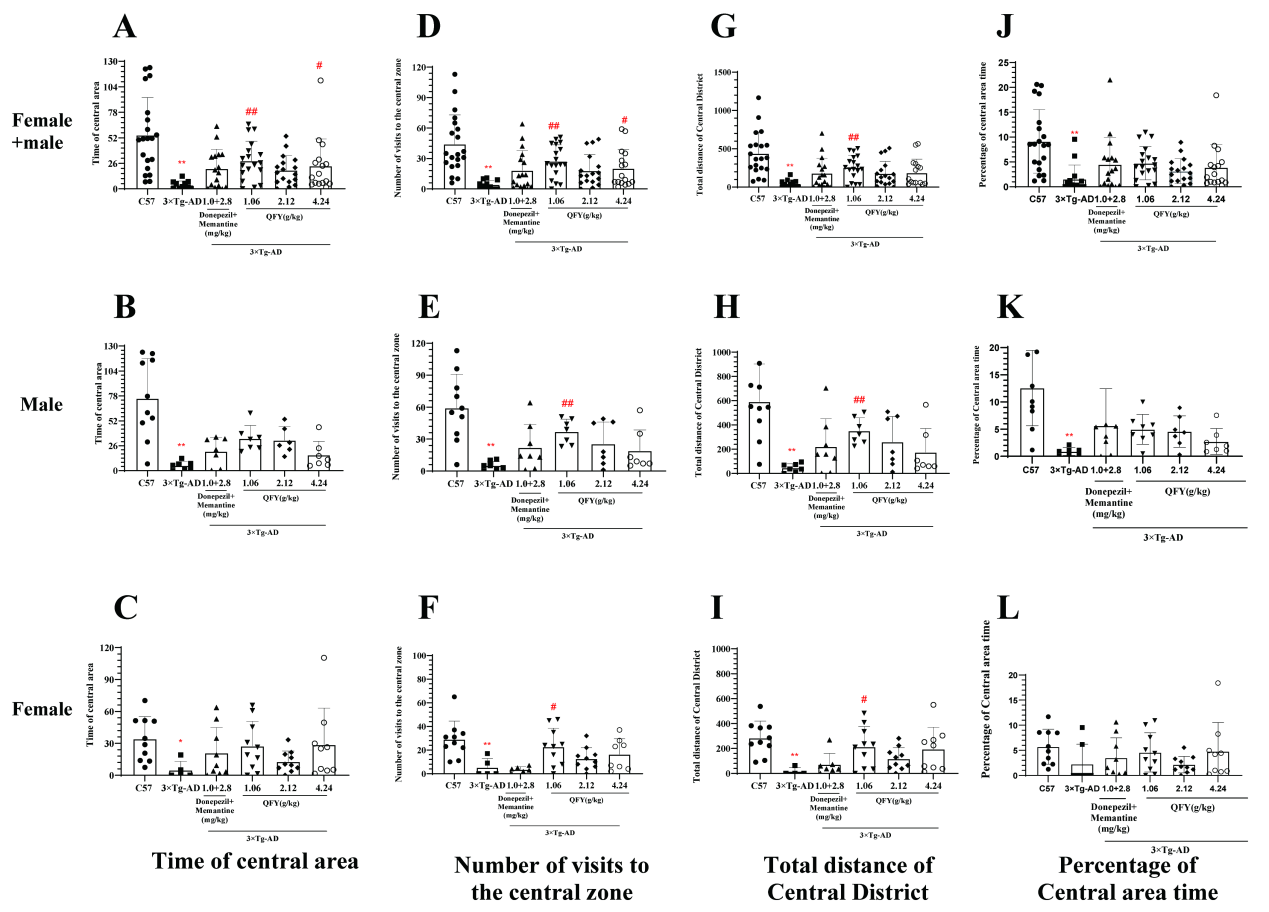


Fig.1 The effect of QFY on anxiety-like behavior of 3×Tg-AD transgenic mice in open field test. A-C, Time spent in central area; D-F, Number of visits to the central zone; G-I, Total distance in central district; J-L, Percentage of central area time. Mean±S.D., n=7-20; ^*^*P*<0.05, ^**^*P*<0.01 vs C57 mice, Student`s *t*-test; ^#^*P*<0.05, ^##^*P*<0.01 vs 3×Tg-AD mice, One-way ANOVA followed by Dunnett’ s multiple comparisons test; GraphPad 8.0.1.


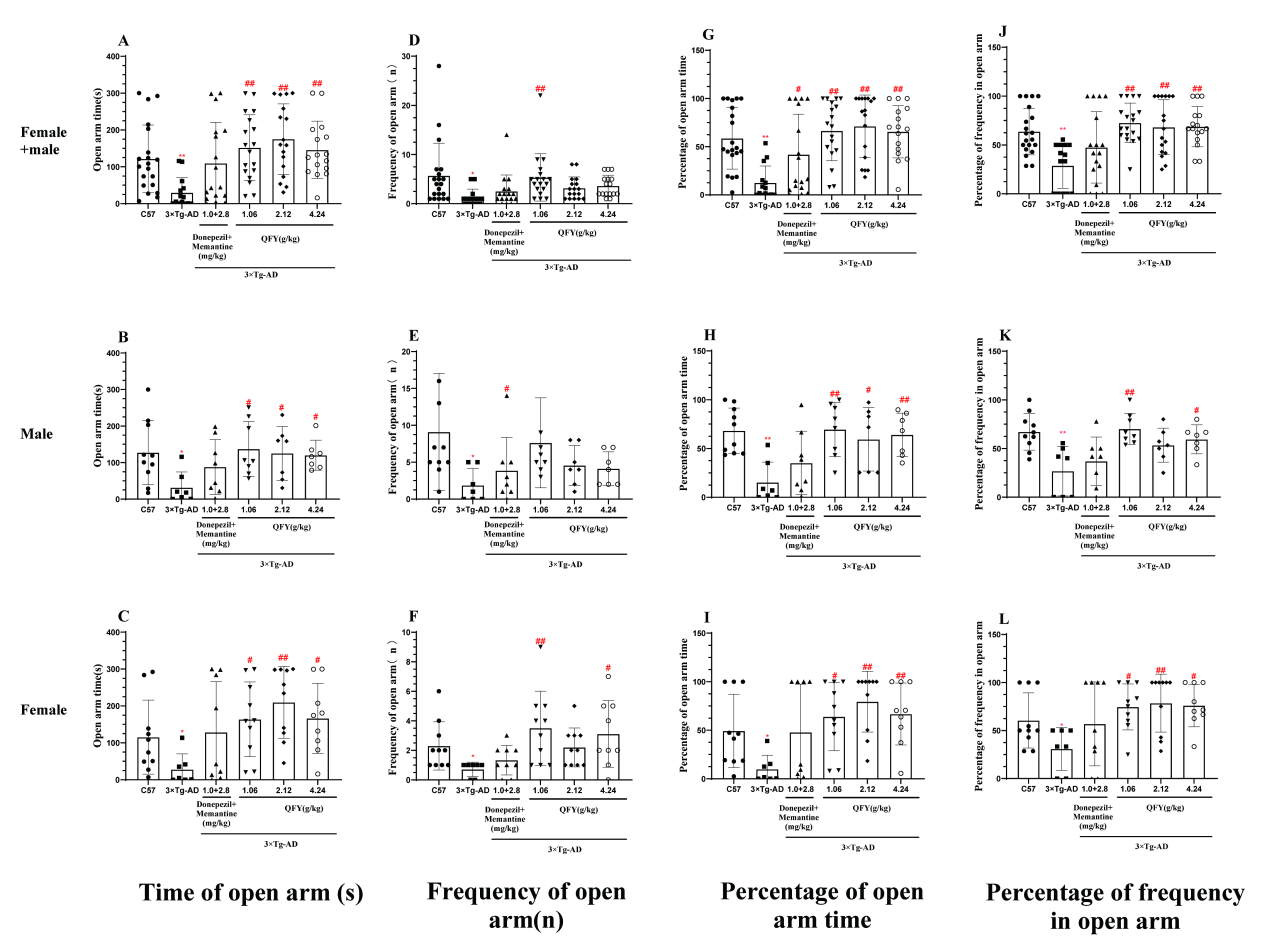


Fig.2 The effect of QFY on anxiety-like behavior of 3×Tg-AD transgenic mice in elevated plus maze test. A、B、C，Time of open arm; D、E、F，Frequency of open arm entry times; G、H、I，Percentage of open arm time; J、K、L，Percentage of frequency in open arm time. Mean±S.D. n=7-9. ^*^*P*<0.05, ^**^*P*<0.01 vs C57 mice, Student`s *t*-test; ^#^*P*<0.05, ^##^*P*<0.01 vs 3×Tg-AD mice, One-way ANOVA followed by Dunnett's multiple comparisons test, GraphPad 8.0.1.


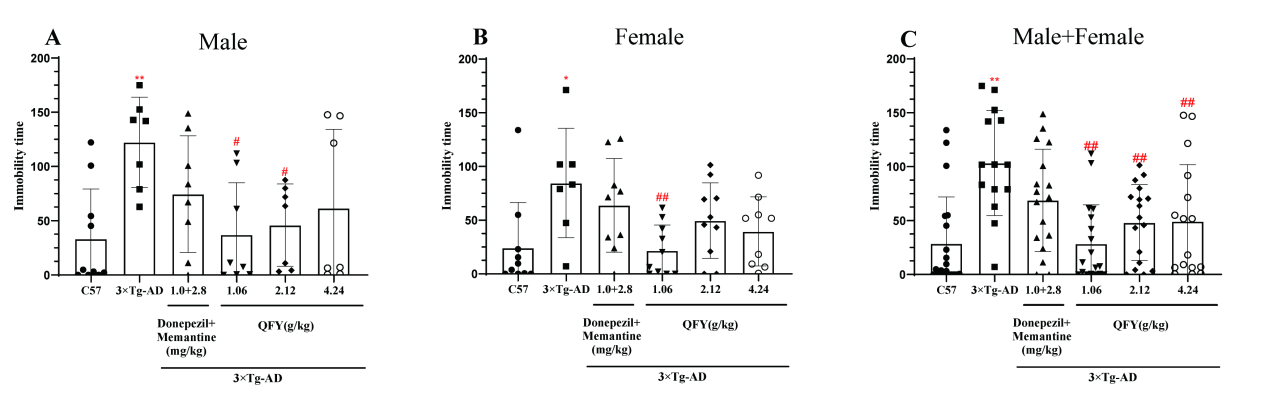


Fig.3 The effect of QFY on depressive-like behavior in 3×Tg-AD transgenic mice in the forced swimming experiment. A, The immobility time of Male mice. B, The immobility time of Female mice. C,The immobility time of male + female mice. Mean±S.D.n=7-9.^*^*P*<0.05, ^**^*P*<0.01 vs C57 mice, Student`s *t*-test; ^#^*P*<0.05, ^##^*P*<0.01 vs 3×Tg-AD mice, One-way ANOVA followed by Dunnett's multiple comparisons test, GraphPad 8.0.1


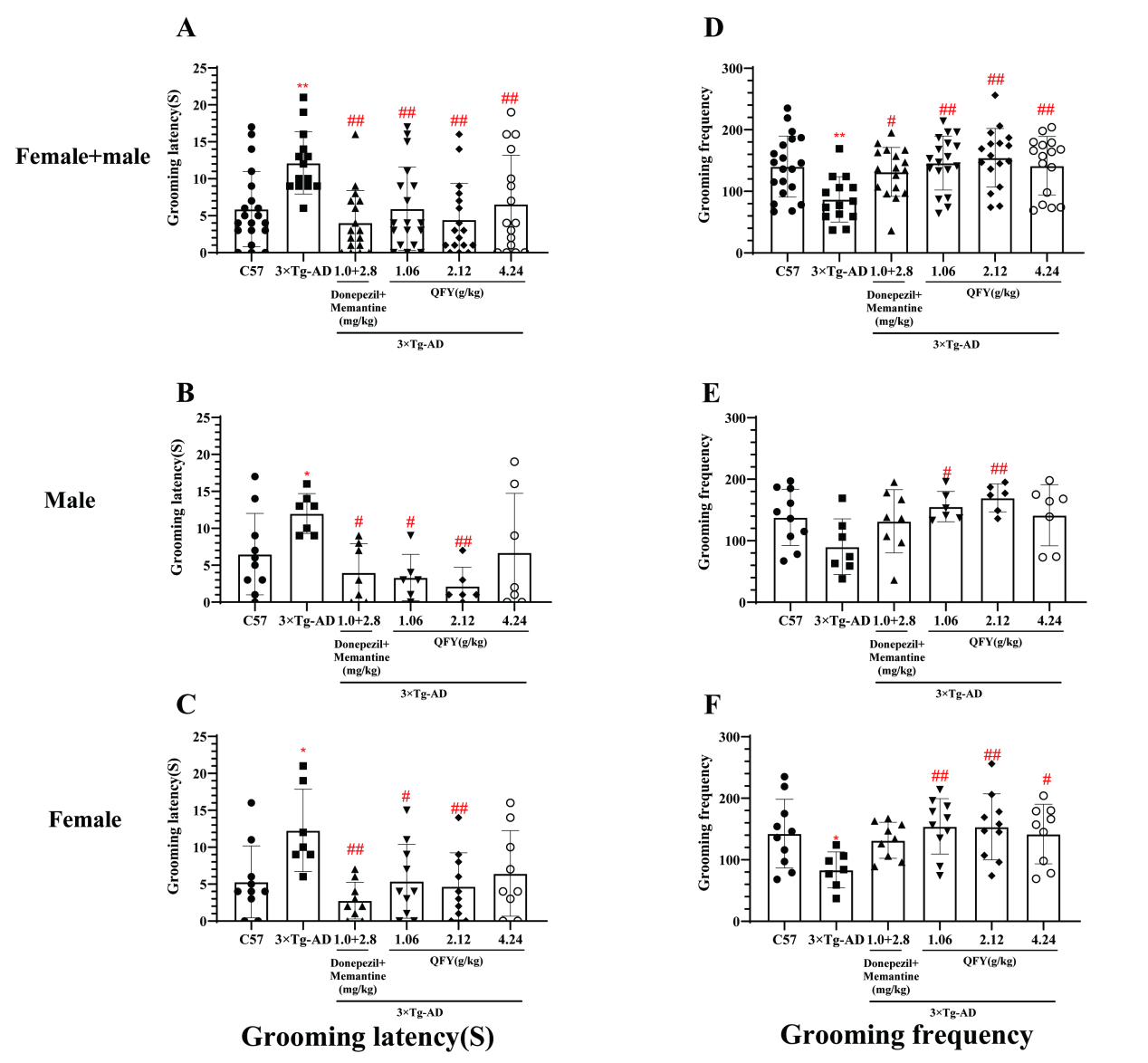


Fig.4 The effect of QFY on depressive-like behavior in 3×Tg-AD transgenic mice in the sugar water splash test. A-C, Grooming latency; D-F, Grooming frequency. Mean±S.D., n=7-20; ^*^*P*<0.05, ^**^*P*<0.01 vs C57 mice, Student`s *t*-test; ^#^*P*<0.05, ^##^*P*<0.01 vs 3×Tg-AD mice, One-way ANOVA followed by Dunnett’s multiple comparisons test; GraphPad 8.0.1.


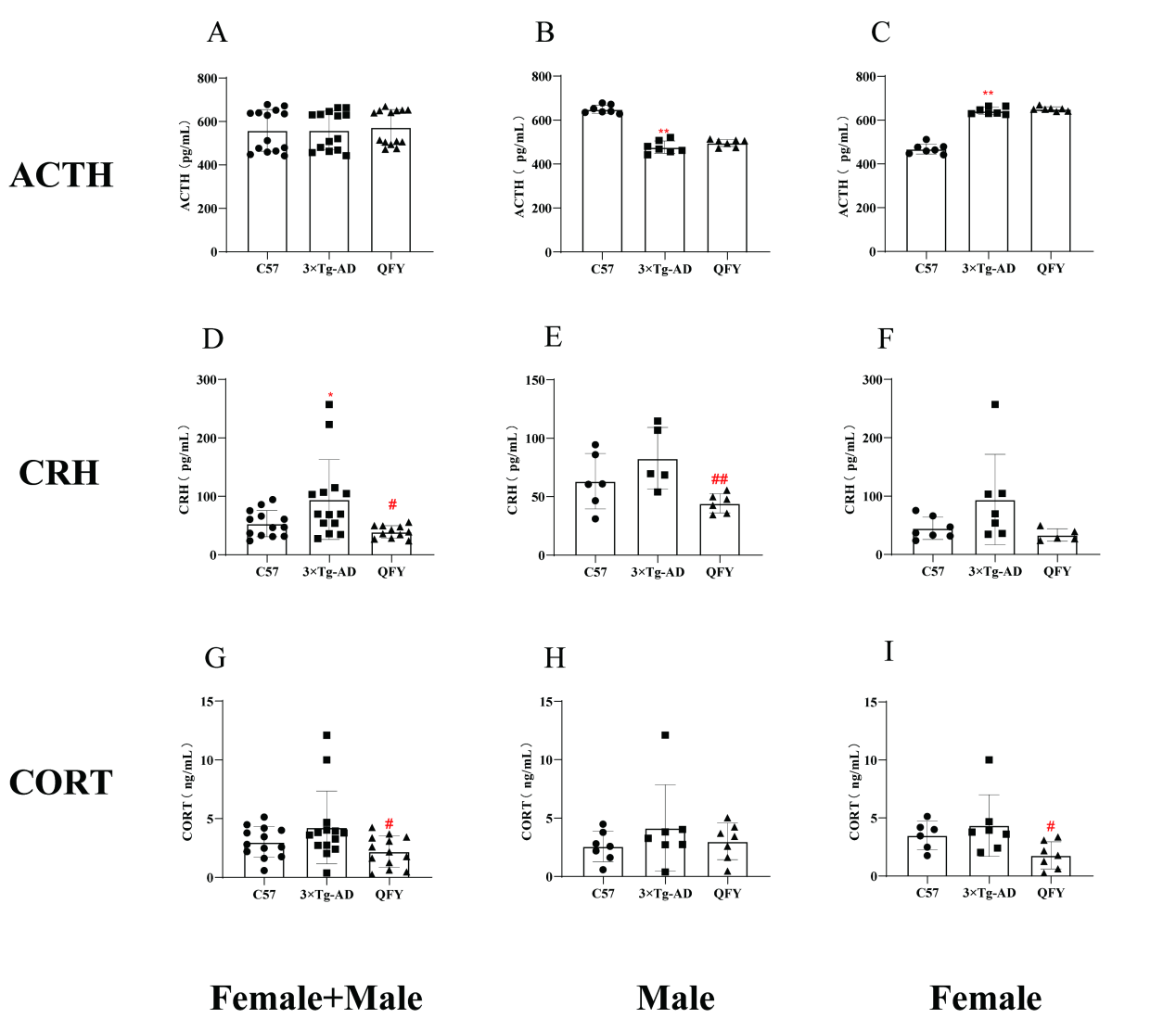


Fig.5 The effect of QFY on hypothalamic–pituitary–adrenal axis of 3×Tg-AD transgenic mice. A-C.The concentration of ACTH in plasma. D-F.The concentration of CRH in plasma. G-I, The concentration of CORT in plasma. Mean±S.D., n=7-14.^*^*P*<0.05,^**^*P*<0.01 vs C57 mice, Student`s *t*-test; ^#^*P*<0.05，^##^*P*<0.01 vs 3×Tg-AD mice, Student`s *t*-test; Graphpad 8.0.1.


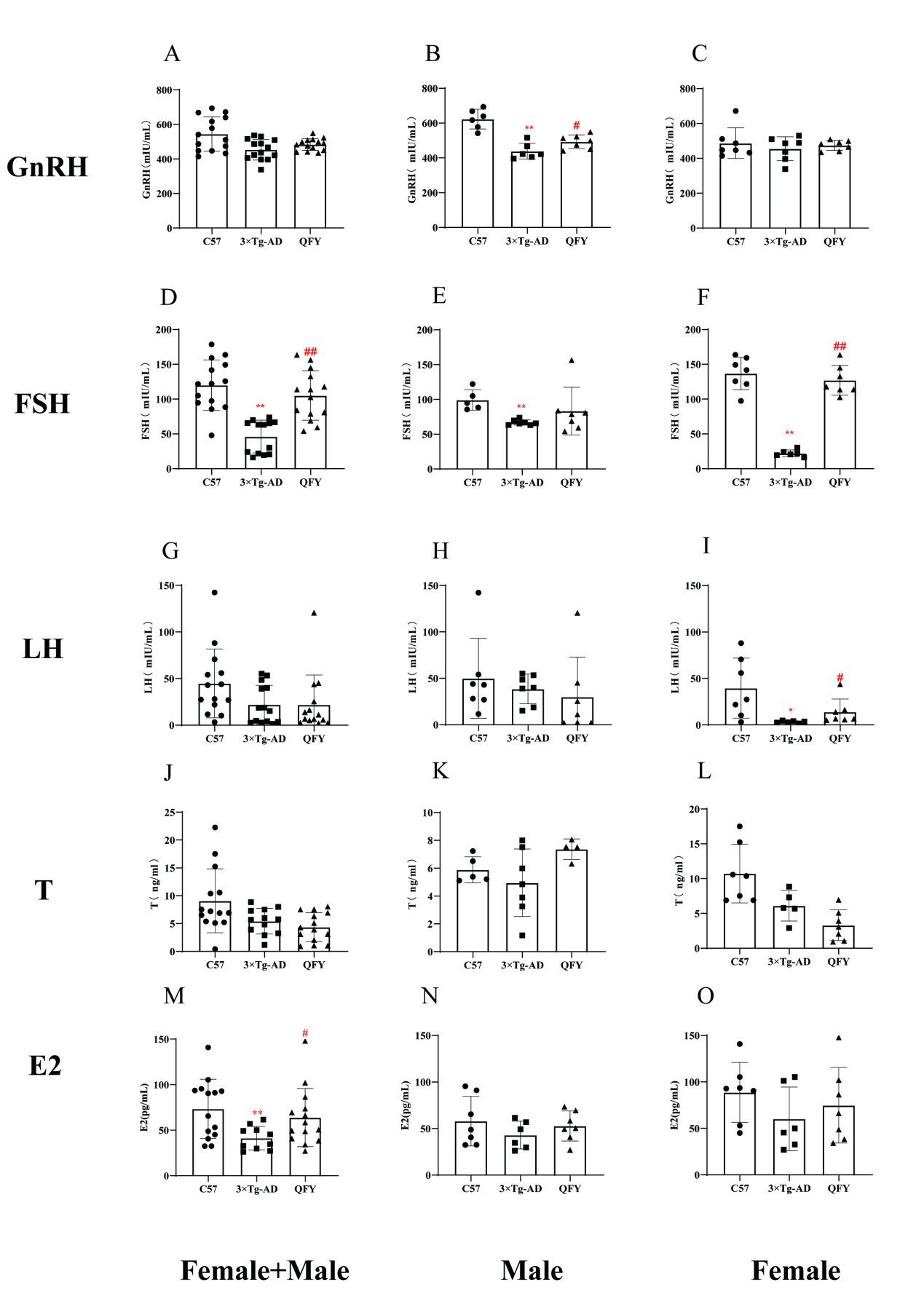


Fig.6 The effect of QFY on hypothalamic-pituitary-gonadal axis of 3×Tg-AD transgenic mice. A-C. The concentration of GnRH in plasma. D-F. The concentration of FSH in plasma. G-I, The concentration of LH in plasma. J-L. The concentration of T in plasma. M-O. The concentration of E2 in plasma. Mean±S.D. n=7-14.^*^*P*<0.05,^**^*P*<0.01 vs C57 mice, Student`s *t*-test; ^#^*P*<0.05，^##^*P*<0.01 vs 3×Tg-AD mice , Student`s *t*-test; Graphpad 8.0.1.
